# Geochemical variability and microbial metabolic functions in oligotrophic sediments exposed to minor seepage

**DOI:** 10.3389/fmicb.2025.1720187

**Published:** 2026-01-21

**Authors:** Ellen Schnabel, Aurèle Vuillemin, Sarah Esser, Lea Griesdorn, André R. Soares, Pål Tore Mørkved, Steffen L. Jørgensen, Alexander J. Probst, Jens Kallmeyer, the PROSPECTOMICS Consortium

**Affiliations:** 1 GFZ Helmholtz Centre for Geosciences, Section Geomicrobiology, Telegrafenberg, Potsdam, Germany; 2 Faculty of Chemistry, Environmental Metagenomics, Research Center One Health Ruhr of the University Alliance Ruhr, University Duisburg-Essen, Universitätsstraße, Essen, Germany; 3 Centre for Water and Environmental Research (ZWU), University of Duisburg-Essen, Universitätsstraße, Essen, Germany; 4 Department of Earth Science, Centre for Deep Sea Research, University of Bergen, Allégaten, Bergen, Norway; 5 Faculty of Biology, University Duisburg-Essen, Essen, Germany

**Keywords:** denitrification, sulfate reduction, Barents Sea, hydrocarbon seepage, metagenomes, Oxford Nanopore Technology (ONT)

## Abstract

Low primary productivity in Barents Sea surface waters and limited nutrient flux to the seafloor favor nitrification and nitrogen fixation in deep waters, resulting in a dearth of organic substrates in local sediments. The addition of labile hydrocarbons naturally occurring through seepage from subsurface reservoirs could promote microbial activity in organic-lean sediments, notably by denitrifying and sulfate-reducing microbes. Using gravity cores from an area with numerous hydrocarbon reservoirs, we document pore water geochemistry, dissolved gas concentrations, and total cell counts supplemented with taxonomic and functional marker gene analyses from metagenomes and metagenome-assembled genomes. We assess the contribution of the subsurface biosphere in producing geochemical gradients in oligotrophic sediments facing different exposure to minor seepage. In pristine seabed, i.e., not affected by hydrocarbon seepage, nitrate and ammonium profiles were consistent with denitrification down to 1 m below seafloor. By contrast, minor hydrocarbon seepage caused very different pore water profiles, which were indicative of more reducing geochemical conditions in the sediment and more advanced consumption of electron acceptors in pore water. Delivery of favorable organic substrates to anaerobic microbes through seepage was reflected in slightly higher cell densities, CH_4_ and CO_2_ concentrations, but appeared to have little impact on community diversity. This could be explained by metabolic versatility across functional guilds, with limited differentiation of sedimentary niches, favoring polyvalent fermenters at the expense of canonical denitrifiers and sulfate reducers. These versatile fermenters exhibited diverse predicted capabilities for nitrate and sulfate reduction combined with hydrocarbon degradation, (homo)acetogenesis, and nitrogen fixation. Our results further indicate that specific clades of homoacetogens (Lokiarchaeia, Bathyarchaeia, and Dehalococcoidia) could support cross-feeding interactions when fueled by simple hydrocarbons through seepage, particularly those associated with dissimilatory sulfur metabolism and fermentation of intermediate metabolites. In the absence of hydrocarbon-derived electron donors, the same clades appear capable of energy-conserving (homo)acetogenic fermentation on organic residues. Thus, we conclude that slow-growing (homo)acetogens that are ubiquitous in the marine subseafloor actively contribute to balancing biogeochemical cycles in oligotrophic sediments impacted by minor hydrocarbon seepage.

## 1 Introduction

More than half of the global seafloor is covered by organic-poor sediments (D’Hondt et al., 2004), creating a low-energy environment. Nevertheless, the largest microbial ecosystem on Earth sustains metabolically diverse microbial communities that play a crucial role in global biogeochemical cycles (Joye et al., 2022). In marine sediments, the primary microbial activity is heterotrophic oxidation of sedimentary organic matter (OM) via the reduction of various electron acceptors (Froelich et al., 1979). However, primary OM production and sedimentation rates decrease as a function of distance from land, thereby determining microbial population densities (Kallmeyer et al., 2012) and biogeochemical activities (D’Hondt et al., 2019) at the seafloor and below. Although only slowly accumulating, OM persists after burial (Estes et al., 2019) in the form of low-energy oxidized organic residues, i.e., carboxylic acids (LaRowe and Van Cappellen, 2011). Thus, in pelagic sediments, organic carbon breakdown by microbial populations has been predicted to mainly occur through fermentation despite electron acceptor availability in pore waters (Bradley et al., 2020). Consequently, offshore subseafloor ecosystems are populated by selective microbial taxa constituting a deep biosphere facing long-term energy limitation (Orsi et al., 2020). However, marine cold seeps originating from deep sediment layers have the potential to connect the subseafloor and seafloor biosphere (Chakraborty et al., 2020), producing unexpected taxonomic and functional diversity patterns (Ruff et al., 2015). While significant research has been carried out on hydrocarbon (HC) seeps with obvious surface expressions like carbonate mounds (Naeth et al., 2006), much less attention has been paid to inconspicuous or transient seepage.

The Barents Sea is an economically important area, as it contains a large number of HC reservoirs (Dore and Lundin, 1996; Færseth, 2020), which are in production or being commercially prospected since decades (Doré, 1995; Johansen et al., 1993). All HC reservoirs are known to leak to varying extents (Heggland, 1998), influencing subsurface ecosystems by adding HC-derived electron donors to the overlying sediments. HC migration pathways can vary considerably due to variable leakage rates and volumes (Argentino et al., 2021), concentration gradients, mass flow directions, and near-surface processes, such as carbonate precipitation (Himmler et al., 2024). All these temporally and spatially varying factors play a key role in controlling near-surface geochemical and biological reactions (Abrams, 2020), and thereby can be expected to alter microbial density, diversity and/or metabolic functions. Whether HCs reach the sediment surface or undergo complete mineralization defines the degree of visible seepage manifestations at the sediment–water interface (SWI).

From the ocean surface to subseafloor, microbial transformation processes are key to marine ecosystems as they control vertical export of nutrients and productivity in the deep ocean (Zehr and Kudela, 2011). In organic-rich continental margin sediments, denitrification usually prevails followed by sulfate reduction (Vuillemin et al., 2022; Vuillemin, 2023). In oligotrophic pelagic sediments, nitrification appear to be more quantitatively important (Reese et al., 2018; Vuillemin et al., 2019). By introducing significant amounts of HCs into marine sediments, active seeps create unusual microbial habitats acting as hotspots of carbon cycling in the otherwise organic-lean subseafloor (Joye et al., 2010). While large HC leakages result in obvious seeps with conspicuous manifestations at the seafloor (Serov et al., 2023), minor seeps are characterized by low and diffusive HC fluxes that remain inconspicuous (Schnabel et al., 2025a). In the former case, continuous HC seeps result in clear surface expressions, such as seabed oil mats and ocean surface slicks (Amindzadeh et al., 2013; Serov et al., 2023), gas ebullition with chimneys, pockmarks, flares (Schroot et al., 2005; Serov et al., 2024) and methane-derived carbonate crusts at the seafloor (Argentino et al., 2022). In the latter case, light and condensate HCs disperse intermittently, mostly via diffusion, and are expected to be fully degraded before reaching the SWI. Furthermore, the methane detected at the ocean surface represents a mixture of thermogenic and biogenic gases (Pankratova et al., 2022), including releases from methane hydrates (Vadakkepuliyambatta et al., 2017; Argentino et al., 2021). Although minor seepage triggers biogeochemical processes known to promote variable mineral properties (Schnabel et al., 2025b) and pore water geochemistry (Schnabel et al., 2025a), changes in microbial community composition and activity are often overlooked (Abrams, 2020).

Our study particularly aims to quantify the impact of minor HC seepage on microbial populations, their metabolic functions and surrounding biogeochemical conditions in oligotrophic sediments of the Barents Sea (Knies and Martinez, 2009). For this, we combine pore water geochemistry, dissolved gas concentrations, and total cell counts with short- and long-read metagenomic sequencing and a catalog of functional marker genes from metagenome-assembled genomes (MAGs). We determine how the taxonomic and functional diversity of the subsurface biosphere respond to variable exposure to HC seepage. Based on our comprehensive dataset, we advocate the implementation of metagenomics to decipher subtle variations in geochemical gradients inherent to versatile redox processes that govern biogeochemical cycles in the context of minor HC seepage compared to pristine seabed.

## 2 Methods

### 2.1 Study area and sampling procedures

**Figure 1:**
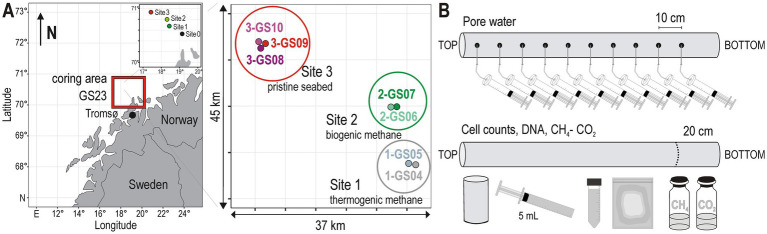
Locations selected for sediment gravity coring in the Barents Sea, and sampling scheme. **(A)** Location of the sampling area GS23 (square) with close-up to the four coring sites presenting oil (Site 0), thermogenic (Site 1), and biogenic gas (Site 2) anomalies, and pristine seabed as reference (Site 3). **(B)** Gravity cores were aliquoted for pore water extraction, total cell counts, DNA extraction, and dissolved gas analyses.

Cruise no. 248 of the Research Vessel *G. O. Sars* (GS23) covered a transect from Tromsø to Svalbard from June 6th to 18th 2023. Sediment samples were collected from three locations (Figure 1A) in the southern Barents Sea (70°31′-70°55’ N / 17°25′-19°08′E), using a 5 m-long gravity corer. Sampling sites were selected based on previous in-depth surveys of seafloor pockmarks and gas leaks (Rise et al., 2015). The sampling area (Håkjerringsdjupet) is located south of the HC fields in the Hammerfest Basin (Hansen, 2017; Matapour et al., 2018) and features dense pockmarks indicative of present and/or past fluid expulsion (Rise et al., 2015). A total of seven gravity cores (length: 0.5–3 m; diameter: 10 cm) were retrieved from two sites with potential HC seepage (Site 1 and Site 2), and from one reference site (Site 3). Gravity coring at the site exhibiting oil anomalies (Site 0) was unsuccessful due to the presence of a carbonate hard ground (not shown). Site 1 (1-GS04, 1-GS05) and Site 2 (2-GS06, 2-GS07), which are ca. 5 km apart (Figure 1A), have known underlying thermogenic and biogenic gas anomalies, respectively, while Site 3 (3-GS08, 3-GS09, 3-GS10) is located on pristine seabed, unaffected by HC seepage, ca. 20 km to the northwest (Supplementary Figure S1). The seven gravity cores were sampled on board immediately after recovery for various types of analyses (Figure 1B).

Oxygen concentrations were measured using an optode connected to a MICROX TX3 single-channel fiber-optic oxygen meter (PreSens, Regensburg, Germany), as previously published (Zhao et al., 2023). The optodes were inserted into holes drilled in the core liners for pore water extraction. Oxygen concentrations were systematically zero at 5 cmbsf. Pore water was then extracted by inserting Rhizons (Rhizosphere Research Products, Wageningen, The Netherlands) into the sediment through holes drilled in the core liners every 10 cm (Figure 1B). About 5 mL of pore water were collected in 24 h, subsequently filtered (PES filter, pore size 0.2 μm, Labsolute, Germany) and aliquoted as follows: 1.5 mL without further treatment for anion and cation measurements; 2 mL in a glass vial amended with 50 μL saturated HgCl₂ to inhibit bacterial activity (Edenborn et al., 1985) and closed without headspace for alkalinity measurements; 1.5 mL mixed with 200 μL ZnCl₂ (20% w/v) to precipitate sulfide as ZnS for hydrogen sulfide (H₂S); 3 mL frozen at −18 °C for dissolved P and N species analysis (PO_4_^3−^, NO_2_^−^, NO_3_^−^, NH_4_^+^). All other pore water samples were stored at 4 °C until analysis.

Sediments from the bottom 20 cm of each gravity core were subsampled for methane (CH_4_) and carbon dioxide (CO_2_) concentrations, total cell counts and DNA extraction. For cores longer than 100 cm, sediment material was also sampled at 50–70 cmbsf for DNA extraction. For dissolved gas concentrations, 3 cm^3^ of sediment were collected using 15 mL cut-off syringes as mini-corers and transferred into 10 mL gas-tight glass vials filled with a saturated NaCl solution. The vials were sealed without headspace, stored upside down, and kept at room temperature for 70 days until analysis in the home lab in Potsdam. For enumeration of total cell counts, 2 cm^3^ of sediment were transferred into a 15 mL centrifuge tube containing 8 mL of NaCl-formalin fixative solution (25 g × L^−1^ NaCl, 20 g × L^−1^ formalin, 2% final concentration), and the mixture homogenized. Sediments were then pushed out of the liner and the outer 1–2 cm scraped off with a sterile spatula to remove possible contamination. The remaining sediment was placed in a gas-tight foil back, flushed with N_2_ gas and frozen at −80 °C for DNA extraction in the home lab in Duisburg-Essen.

### 2.2 Concentrations of dissolved gases and pore water solutes

Prior to gas measurements, 3 mL of helium was introduced into the vials as headspace and allowed to completely equilibrate with the dissolved CH_4_ and CO_2_ by placing the vials on a shaker at 300 rpm for 24 h. Any remaining clay aggregates were broken down by vortexing, and 250 μL of the headspace gas were injected into an Agilent 7890A gas chromatograph (GC) equipped with an HP-PLOT Q column (all Agilent, Santa Clara, United States). The device was set to 13 psi steady pressure, 17.2 mL × min^−1^ column flow, and 50 °C oven temperature. System calibration was performed using 250 μL of pure analytical standards with spiked CH_4_ and CO_2_ concentrations of 100 ppm and 5,170 ppm, and 310 ppm and 5,270 ppm, respectively. Initial concentrations were converted from ppm to micromolar (μmol × L^−1^) by applying the ideal gas law.

Pore water anion concentrations were determined using a suppressed ion chromatography (IC) system equipped with a SykroGel A × 300 AB-A01 column (all Sykam GmbH, Fürstenfeldbruck, Germany). Due to high salt concentrations in the pore water that could potentially overload the instrument and result in inaccurate measurements, we diluted all samples 1:40 with MilliQ water before injection. The eluent contained 7.3 mg × L^−1^ NaSCN and 636 mg × L^−1^ NaCO_3_. The pump rate was set to 1 mL × min^−1^, and the injected sample volume was 50 μL. A multi-element anion standard (Sykam) was measured every 10 samples. Based on a respective signal-to-noise (S/N) ratio of 3 and 10, the calculated detection (S/N = 3) and quantification limits (S/N = 10) are as follows: Cl^−^ (5.7 μM; 16.17 μM) and SO_4_^2−^ (2 μM; 8.4 μM). All samples were measured in triplicates, and the results averaged. The average standard deviation of three technical replicates was better than 3%.

Pore water cation concentrations were measured on a non-suppressed IC system (all Sykam) equipped with a Maisch ReproSil 100 Cat column (Dr. Maisch Chromatographie, Ammerbuch-Entringen, Germany), run with an eluent consisting of 120 μL × L^−1^ methane sulfonic acid and 175 mg × L^−1^ 18-Crown-6 ether (i.e., 1,4,7,10,13,16-hexaoxacyclooctadecane). The flow rate was set to 1.2 mL × min^−1^. For each triplicate, we injected 10 μL of sample previously diluted 1:40 with MilliQ water. A Cation Multi-Element Standard (Carl Roth, Karlsruhe, Germany) was diluted 5 times for calibration. The calculated detection (S/N = 3) and quantification (S/N = 10) limits are as follows: Na^+^ (5.8 μM; 35 μM), K^+^ (9.1 μM; 54.7 μM), Mg^2+^ (9.6 μM; 44.6 μM), and Ca^2+^ (8.3 μM; 38.5 μM). The average standard deviation of measurements of the triplicates was better than 3%.

Alkalinity was determined via titration, using the Visocolor HE alkalinity AL 7 kit (Macherey-Nagel GmbH, Düren, Germany), and adapted to the small sample volume (Schnabel et al., 2025a). The detection limit is 0.2 mEq × L^−1^ and triplicate measurements differed by less than 3%.

Concentrations of ammonium (NH_4_^+^), nitrate (NO_3_^−^), and nitrite (NO_2_^−^) were determined photometrically by applying the indophenol method, and the non-reduced and Cu-Cd coil-reduced N-1-naphthylethylenediamine dihydrochloride and sulfanilamide methods, respectively, as published (Zhao et al., 2023). The colored solutions were analyzed on a QuAAtro continuous flow analyzer (SEAL Analytical Ltd., Southampton, UK), following the manufacturer’s protocol. Dissolved PO_4_^3−^ concentrations were determined photometrically on acidified pore water samples using the molybdate reagent and measuring the colored solution absorbance (Hansen and Koroleff, 1999).

### 2.3 Total cell counts

Cell counts were performed using a protocol based on Kallmeyer et al. (2008). The fixed samples were diluted 100-fold in 25 g × L^−1^ NaCl solution, and 25 μL of slurry were evenly distributed onto black 0.2 μm polycarbonate Cyclopore membrane filters (Whatman International Ltd., Maidstone, UK) using a vacuum filtration system. Cells on the filter were stained using a solution composed of SYBR Green I (10 μL) (Molecular Probes, Eugene OR, United States), phenylenediamine (100 μL), glycerol (300 μL), MilliQ water (300 μL), and VECTASHIELD^®^ Antifade Mounting Medium H-1000-10 (300 μL) (Vector Laboratories, Burlingame CA, United States). Cell staining was performed by applying 15 μL of staining solution to the filter, and the total number of cells was counted by examining 200 fields of view under an epifluorescence microscope (Leica DM2000, Wetzlar, Germany). Total cell concentrations were calculated per sediment volume (log_10_ cells × cm^−3^) from triplicate measurements, with standard deviations below 15%.

### 2.4 DNA extraction, metagenome sequencing, and read processing

Total DNA was extracted from 10 g of sediment per reaction, using the DNeasy PowerSoil Pro Kit (Qiagen, Hilden, Germany) and following the manufacturer’s instructions. Library preparation and metagenomes were successfully generated, using the Nextera XT DNA Library Preparation kit (Illumina, San Diego, United-States), for five DNA extracts out of the nine initially sent. Sequencing data were obtained from the following samples: gravity core 1-GS05 (50–70 and 253–273 cmbsf); gravity core 2-GS06 (31–51 cmbsf) and 2-GS07 (36–55 cmbsf); and gravity core 3-GS09 (118–138 cmbsf). Sequencing was performed on a NovaSeq 6,000 Illumina platform at CeGaT GmbH (Tübingen, Germany), aiming for 30 Giga base pairs (bps) per sample (reads: 2 × 150 bps).

In addition, Oxford Nanopore Technology (ONT) sequencing was performed in-house at the Probst Lab, University of Duisburg-Essen. Nanopore sequencing libraries were prepared from replicate DNA extracts, applying the protocol’s last update (24/04/2023) of the Ligation sequencing gDNA (SQK-LSK109XL) kit with the Native Barcoding Kit 24 V14 according to the manufacturer’s manual, with some modifications: after addition of the Native Adapter (NA) and T4 DNA Ligase with the Quick Ligation™ Kit (New England BioLabs GmbH, Frankfurt am Main, Germany) to the pool of barcoded samples, reactions were stored overnight at 4 °C (instead of 20 min at room temperature), which improved the yield of DNA after clean-up; incubation times on the Hula Mixer were doubled during the whole protocol in order to improve DNA binding to magnetic beads; due to low DNA content, the sample amount was not reduced to an equimolar mass for the Native barcode ligation step in order to keep the nanopores of the flow cell functional during sequencing (Simon et al., 2023). The barcoded samples were sequenced over 72 h until complete exhaustion of the flow cell.

Quality-control for Illumina metagenomes included the removal of PhiX sequencing control, Illumina adapter sequences, and sequencing artifacts as well as quality filtering of reads with bbmap (Bushnell, 2014) along with a windowed adaptive trimming with sickle (Joshi and Fass, 2011). Nanopore raw signal was base-called, using dorado (simplex) setting the minimum quality of individual reads to ≥10 (−min_qscore 10), as recommended to optimize later hybrid assemblies. Quality control of ONT reads was performed using Filtlong^1^ to remove fragments smaller than 1,000 bps and retrieve only the 95% best reads. Hybrid assembly of short and long reads was achieved using metaSPAdes (Nurk et al., 2017) in a high-performance Ubuntu Linux server with 1.15 TB RAM using 40 CPU threads, keeping scaffolds longer than 1,000 bps. Illumina reads were mapped against hybridly assembled scaffolds with bowtie2 (Langmead and Salzberg, 2012) to retrieve the mean coverages of each scaffold, after which their GC % content and length were calculated, using in-house python scripts. Gene prediction was performed with Prodigal (Hyatt et al., 2010) using flags “-p meta -m,” and the resulting predicted protein sequences annotated against FunTaxDB v. 1.4 using uBin (Bornemann et al., 2023).

Gene-level FunTaxDB taxonomic annotations were used to find the consensus taxonomy of each scaffold (Bornemann et al., 2023), after which an overview file was generated per assembly of the GC %, length, coverage and taxonomy of each scaffold. Single-copy genes (SCGs) were predicted for each scaffold (Probst et al., 2017). Automated binning into metagenome-assembled genomes (MAGs) was performed using differential coverage information for each scaffold across all samples (as calculated with bowtie2) and using MaxBin2 (Wu et al., 2016) for identification of the 40 and 107 marker gene sets, metaBAT2 (Kang et al., 2019) and SemiBin2 (Pan et al., 2023). The optimized set of bins per sample was then calculated with DAS_Tool (Sieber et al., 2018), manually curated with uBin (Bornemann et al., 2023) using information collected in the overview file and SCG information. Bin quality and taxonomy were inferred with CheckM2 (Chklovski et al., 2023) and GTDB-tk against the database v. r220 (Chaumeil et al., 2020), respectively. Dereplication was performed with galah^2^ at 95% Average Nucleotide Identity (ANI) (Aroney et al., 2024), using the bin quality report produced by CheckM2. The relative abundance of each MAG was assessed by plotting the total number of mapped reads normalized to their respective genome size via coverM module “genome” (Aroney et al., 2025).

A phylogenetic tree of the MAGs was computed based on 16 concatenated ribosomal protein sequences against a selection of closely related representative MAGs from the GTDB database (Parks et al., 2022), according to published scripts (Graham and Tully, 2018). The computed tree was visualized using iTOL (Letunic and Bork, 2024).

The ribosomal protein S3 (*rpS3*) marker gene was used to estimate the prokaryotic community composition diversity and account for low-abundant microbial taxa in complex communities (Sharon et al., 2015). Marker genes were identified with species-specific Hidden Markov Models (HMMs) and by comparing the amino acid sequences of predicted genes against the UniRef100 database (23/06/2021) (Suzek et al., 2015) using BLASTp with DIAMOND protein aligner (Buchfink et al., 2021). The *rpS3* gene nucleotide sequences with 1,000 bps flanking regions were extracted for all samples and clustered with MMSeqs2 (Steinegger and Söding, 2017) in cluster-mode 2, coverage-mode 1, minimum breadth of 95% and minimum sequence identity of 95%. All *rpS3* gene sequences were taxonomically annotated by comparing them with *rpS3* sequences extracted from the GTDB v. 220 (Parks et al., 2022) with USEARCH “-ublast” v. 10.0.240_i86linux64 (Edgar, 2010). Quality-controlled reads of all samples were mapped against their representatives with bowtie2 in sensitive mode (Langmead and Salzberg, 2012). Reads mapping with more than five mismatches were excluded. The mean coverage depth of extended *rpS3* gene sequences was calculated for all sequences with a coverage breadth greater than 95%.

Predicted Open Reading Frames (ORFs) were extracted from both contigs and MAGs, and identified by performing BLASTp searches against the large aggregated genome MetaProt database (Orsi, 2020), using the DIAMOND protein aligner (Buchfink et al., 2015). The database contains 37.8 million predicted proteins compiled from the SEED (www.theseed.org) and NCBI RefSeq databases updated with high-quality MAGs and single-cell assembled genomes (SAGs) from the NCBI protein database. These results were cross-checked, using MetaCerberus (Figueroa III et al., 2024). We selected ORFs encoding proteins involved in microbial processes of sediment nitrogen cycling, namely nitrogen fixation (*nif*, nitrogenase, dinitrogenase), nitrification (*amo*), denitrification (*nar*, *nap*, *nir*, *nor*, *nos*), dissimilatory nitrate reduction to ammonium (*nrf*), or DNRA, NH_4_^+^ and NO_3_^−^ transporters, as well as degradation of organic N sources (*CNH*, *NOS*, *nmo*, urease, nitrilase, nitroreductase) and assimilation (ammonia ligase, ammonia lyase, *nifU*-like proteins, *nus*). With regards to degradation of sedimentary organic carbon, we selected ORFs predicted to have enzymatic activity in the benzoyl-CoA pathway (*bzd*) and HC degradation (*HBCR*, *HLD*, *ARHD*, *DODA*, *BDH*, *EBDH*), as well as those involved in substrate-level phosphorylation (SLP) of volatile fatty acids (VFAs) (*pta*, *akn*, *bkn*, *pkn*), anaerobic fermentation (*LDH*, *pdh*), anaplerosis (*pccAB*) and acetogenesis (*fdh*, *codh*, *cdhA*, *acs*, *acss2*). Microbial processes of sedimentary sulfur cycling were assessed by selecting ORFs encoding enzymes for sulfate uptake (*APS*, *APS-kn*), dissimilatory and assimilatory sulfur metabolism (*apr*, *dsr*, *psr*, *hyd*, *dmso*, *Cys I*), sulfide dehydrogenase (*sud*), sulfate (SO_4_^2−^) and sulfite (SO_3_^2−^) transporters. Fe-related genes present in contigs and MAGs were identified using the curated Hidden Markov Models (HMMs) implemented in the FeGenie pipeline (Garber et al., 2020), with default parameters.

Taxonomic and functional diversity of the contigs was assessed by extracting ORFs encoding the RNA polymerase sigma 70 factor (*RpoD*) for Bacteria and transcription initiation factor IIB (*TFIIB*) for Archaea, as well as ORFs encoding subunits of the ammonia monooxygenase (*amoBC*), respiratory nitrate reductase (*narGH*), periplasmic nitrate reductase (*napA*), copper-containing nitrite reductase (*nirK*), adenylyl-sulfate reductase (*aprAB*), dissimilatory sulfite reductase (*dsrAB*), nitrogen fixation proteins (*nifBHUX*), and benzoyl-CoA reductase (*bzdNOQ*). Phylogenetic analyses of the conserved amino acid alignments of the predicted proteins were conducted in SeaView v.5.0.5 (Gouy et al., 2010). Conserved regions of the alignments were selected using Gblocks with the following settings: allowing for smaller final blocks, gap positions within the final blocks and less strict flanking positions. Phylogenetic trees were computed using PhyLM maximum likelihood (Guindon et al., 2010), with BLOSUM62 as the evolutionary model and 100 bootstrap replicates, and visualized using iTOL (Letunic and Bork, 2024).

The full list of enzymes and gene abbreviations is available as supplement (Supplementary Table S1). All BLASTp results, MAG metadata and *rpS3* gene diversity are available as supplement (Supplementary Data).

## 3 Results

### 3.1 Pore water geochemistry, dissolved gases, and total cell counts

**Figure 2:**
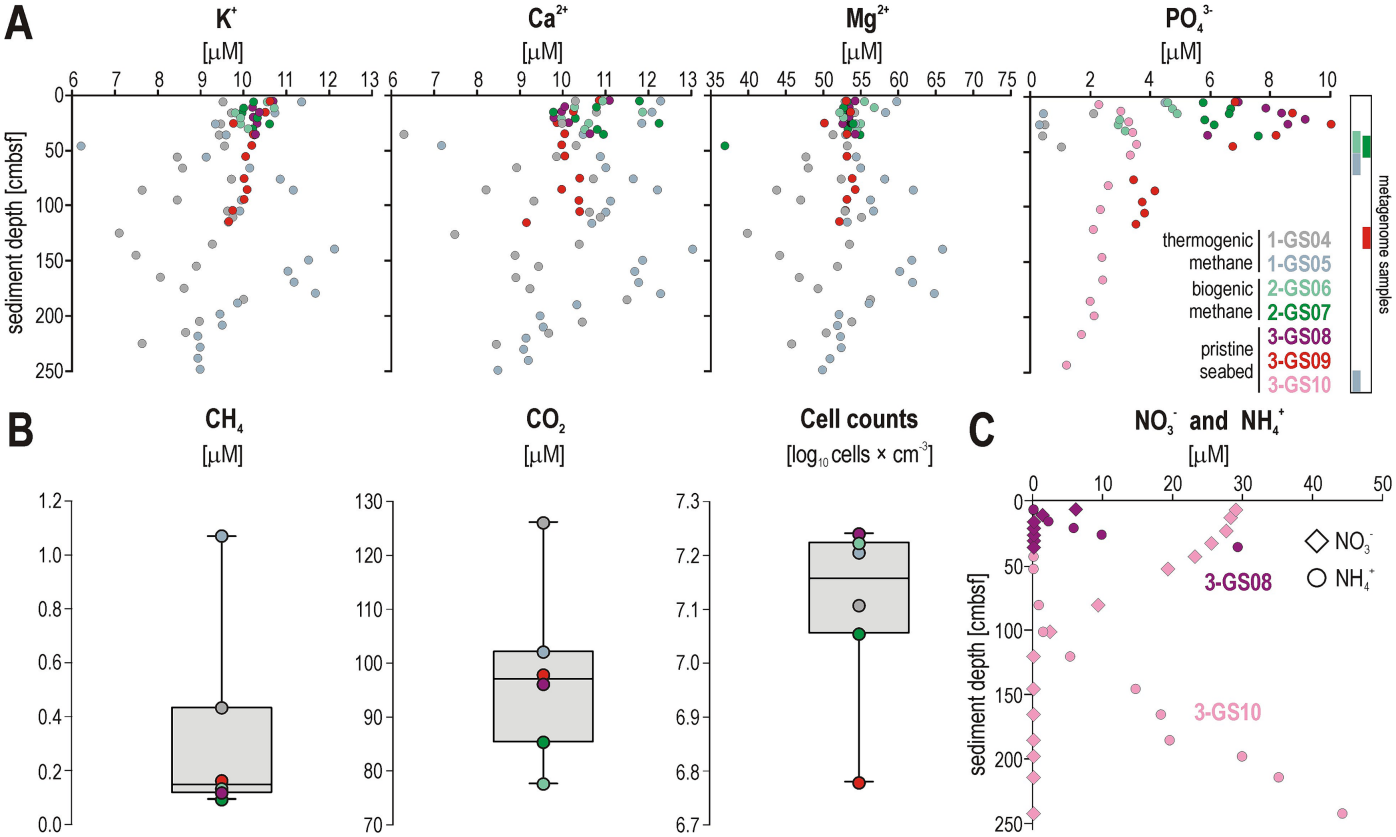
Downcore profiles for major ion concentrations in pore water, dissolved gas concentrations, and cell densities. **(A)** Pore water profiles for potassium (K^+^), calcium (Ca^2+^), magnesium (Mg^2+^), and phosphate (PO_4_^3−^) concentrations in [μM]. **(B)** Point concentrations of methane (CH_4_) and carbon dioxide (CO_2_) in [μM], and total cell counts in [log_10_ cells × cm^−3^] in bottom core sediments. **(C)** Pore water profiles for nitrate (NO_3_^−^) and ammonium (NH_4_^+^) in the two gravity cores from Site 3 where pore water nitrate was measurable. The convex-down nitrate profiles are consistent with denitrification in sediments.

The pore water profiles for major cations (Ca^2+^, Mg^2+^, K^+^) exhibit patterns that tend to covary across solute species within each individual core, but differ across the different cores and sampling sites (Figure 2A). At the SWI, the measured concentrations roughly match seawater concentrations (i.e., Ca^2+^ 13 μM; Mg^2+^ 55 μM; K^+^ 12 μM), whereas variations in downcore profiles could be interpreted in terms of changes in sediment lithologies across sampling sites, such as clay content. Pore water chloride (Cl^−^) fluctuates between 440 and 480 μM, with concentrations lower than seawater (i.e., 545–550 μM), with no discernible downcore trend (not shown).

Phosphate (PO_4_^3−^) concentrations vary across sampling sites (Figure 2A), with lowest concentrations (<2 μM) in the upper parts of cores 1-GS04 and 1-GS05 (Site 1), followed by 2-GS06 (~4 μM) and 2-GS07 (Site 2), while cores 3-GS08 and 3-GS09 (Site 3) display highest concentrations with an increase from 5 to 10 μM in the upper 50 cmbsf, decreasing below. In core 3-GS10 (Site 3), PO_4_^3−^ concentrations decrease steadily down to 1 μM at 250 cmbsf. Such increase and subsequent decrease can be interpreted as pore water release and uptake of PO_4_^3−^ during OM mineralization in surface sediments.

Dissolved CH_4_ concentrations are generally low in all cores (<0.2 μmol × L^−1^), with highest values at Site 1 in the bottom part of core 1-GS05 (1.2 μmol × L^−1^). CO_2_ concentrations are also low, varying between 70 and 140 μmol × L^−1^, with highest values incore 1-GS04 and 1-GS05 from Site 1 (Figure 2B). In the bottom part of all cores, total cell counts are about 10^7^ cells × cm^−3^ of sediment, independent of sampling depth (Figure 2B).

Pore water NO_3_^−^ and NO_2_^−^ concentrations at Site 1 and Site 2 are below the detection limit (1.0 and 0.5 μM for NO_3_^−^ and NO_2_^−^, respectively). In comparison, in gravity core 3-GS08 and 3-GS10 from Site 3, NO_3_^−^ was detected in the uppermost sediments down to 10 and 100 cmbsf, respectively (Figure 2C). Their convex-down profiles are consistent with consumption and thus denitrification in sediments at Site 3.

**Figure 3:**
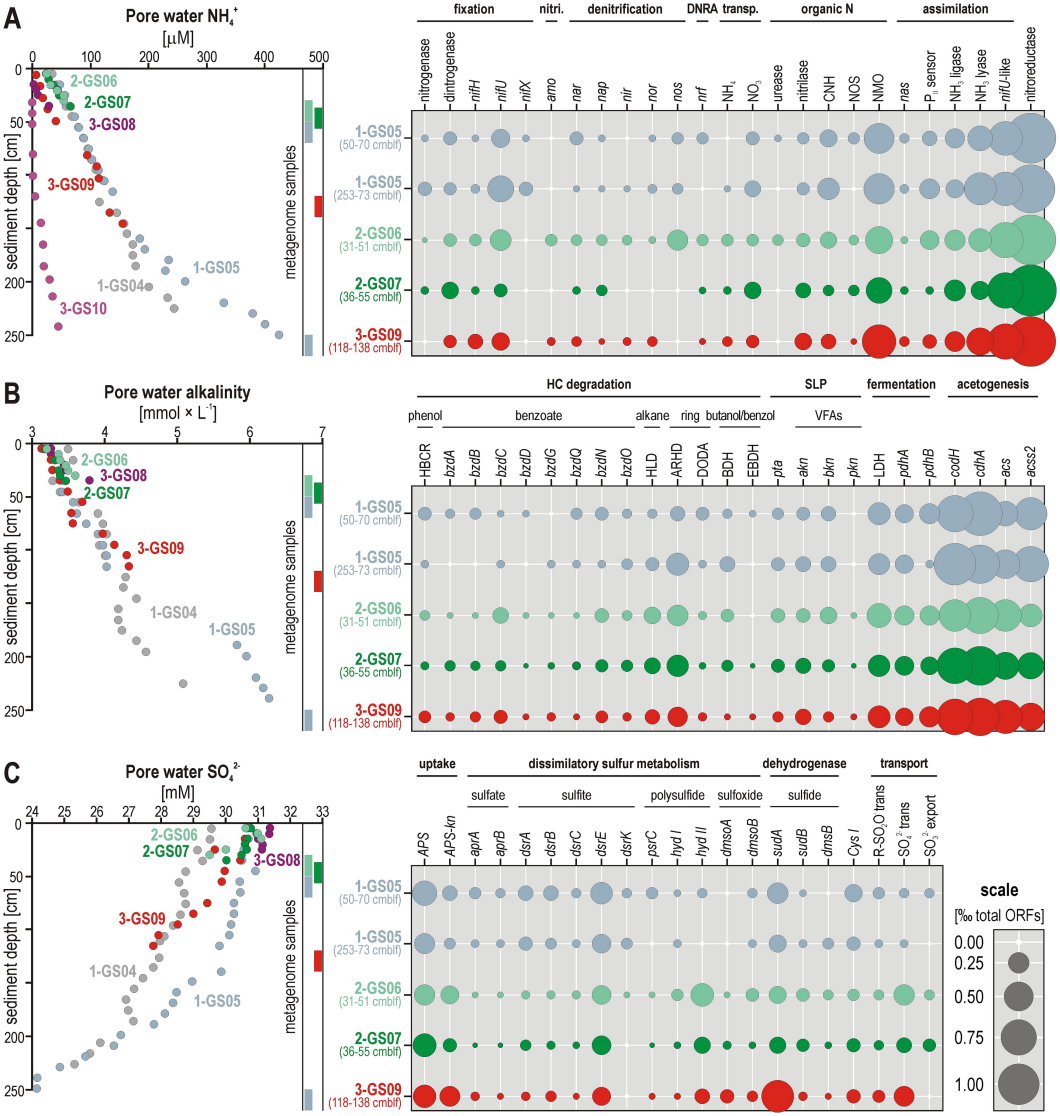
Pore water geochemical profiles and relative abundances of functional marker genes. **(A)** Pore water ammonium (NH_4_^+^) concentrations with relative abundances of functional marker genes related to nitrogen cycling (e.g., fixation, denitrification, ammonification). **(B)** Pore water alkalinity with relative abundances of functional marker genes related to hydrocarbon degradation and carbon cycling (e.g., benzoate anaerobic metabolism, substrate-level phosphorylation or SLP, fermentation, and acetogenesis). **(C)** Pore water sulfate (SO_4_^2−^) concentrations with relative abundances of functional marker genes related to sulfur cycling (e.g., dissimilatory sulfate to polysulfide reduction). All marker genes are displayed in relative abundances of ORFs per Illumina-sequenced metagenome [‰ total ORFs]. Enzymatic names and their corresponding gene abbreviations are listed in the supplement (Supplementary Table S1).

By contrast, pore water NH_4_^+^ concentrations steadily increase in all cores from near zero at the sediment–water interface (SWI) to >400 μM down to 2 mblf (Figure 3A). Pore water alkalinity increases in all cores from ca. 3 at the SWI to >6 mmol × L^−1^ in bottom core (Figure 3B). In all cores, pore water sulfate concentrations decrease with depth from 32 to 30 mM at the SWI to 24 mM below 2 mbsf (Figure 3C).

### 3.2 Metabolic potential for sediment biogeochemical cycles

The selection of functional marker genes (Supplementary Table 1) enabled us to assess the relative importance of microbial processes involved in nitrogen, carbon, sulfur, and iron biogeochemical cycles in the sediment, and thus to determine which redox processes and associated metabolic pathways are key to HC degradation and to setting pore water geochemical signatures (Figures 3A–C).

**Figure 4:**
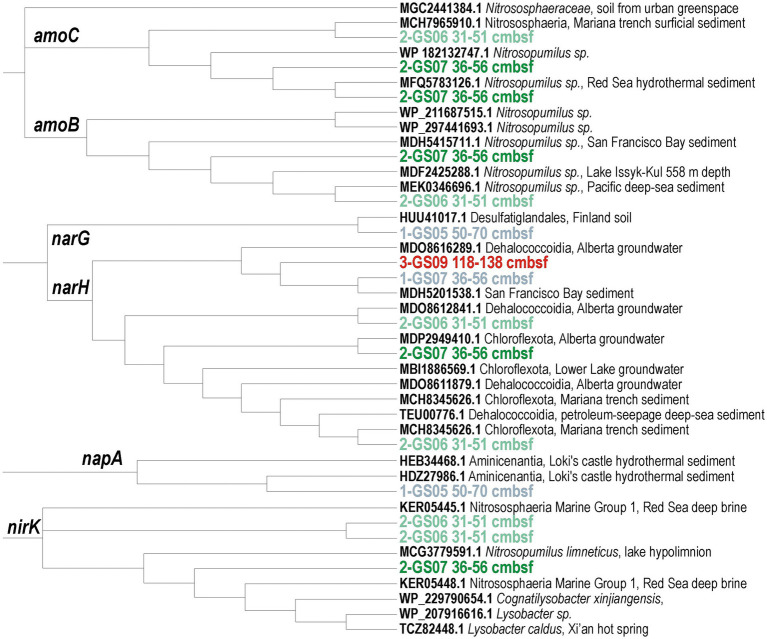
Phylogenetic trees of *amoBC*, *narB-G*, *napA*, and *nirK* protein-encoding genes. PhyML maximum likelihood trees of open reading frames encoding conserved regions of the ammonia monooxygenase subunit beta (*amoB*) and gamma (*amoC*); respiratory nitrate reductase subunit alpha (*narG*) and beta (*narH*); periplasmic nitrate reductase subunit alpha (*napA*); and copper-containing nitrite reductase (*nirK*). Phylogenetic trees are based on 100 bootstrap replicates with BLOSUM62 as the evolutive model. Boldface type signifies sequence accession numbers to the NCBI database.

In terms of nitrogen cycling, most of the marker genes are associated with reduction of N-bearing organic compounds (i.e., nitrilases, nitroreductases, *CNH*, *nmo*) and assimilation of ammonia (i.e., *nifU*-like proteins, NH_3_ ligases and lyases). Heterotrophic denitrification from organic sources appears to prevail as only few predicted genes relate to a complete respiratory denitrification pathway (*nar*, *nap*, *nir*, *nor*), with some rather pointing to (micro)aerobic scavenging of N_2_O (*nos*). Metabolic potential for respiratory nitrate reductase (*nar*, *nap*) and DNRA (*nrf*), which are pathways competing for NO_3_^−^ (Vuillemin, 2023), are equally present. ORFs encoding proteins for N_2_ fixation are relatively abundant (Figure 3A). In comparison, metabolic potential towards nitrification (*amo*, *nxr*) and anammox (*hzo*) is minor, or absent. The pore water profiles demonstrate constant production of NH_4_^+^ during OM breakdown with sediment depth. Phylogenetic analyses of protein conserved regions confirmed metabolic potential for nitrifier denitrification (*amoBC*, *nirK*) by *Nitrosopumilus*-related taxa at Site 2, respiratory nitrate reduction (*narGH*, *napA*) by taxa among the class Dehalococcoidia, Desulfobacteria and Aminicenantia at all three sites (Figure 4). Furthermore, nitrogen fixation potential is common across diverse bacterial and archaeal classes, e.g., Dehaloccoccoidia, Aminicenantia, Lokiarchaeia, and Bathyarchaeia (Supplementary Figure S2).

In terms of HC degradation (Figure 3B), the full benzoyl-CoA reductase pathway (*HBCR*, *bzdA-O*) could be identified. Aromatic HCs (*ARHD*, *DODA*) apparently represent preferential substrates compared to alkanes (*HLD*). Phylogenetic analysis of protein conserved regions encoding the benzoyl-CoA reductase evidences that metabolic capacity to degrade aromatic HCs is common among the classes Anaerolineae, Dehalococcoidia, Lokiarchaeia, and Bathyarchaeia (Supplementary Figure S3). However, the most abundant marker genes related to sediment carbon cycling are functionally assigned to fermentation (*LDH*, *pdhAB*), with SLP of volatile fatty acids (i.e., *pta*, *akn*, *bkn*, *pkn*), complemented by (homo)acetogenesis (*codH*, *cdhA*, *acs*, *acss2*) via the Wood-Ljungdahl pathway (WLP). Diagnostic ORFs predicted to function in biogenic methane production (*mcrA*) was not detected, suggesting that methanogenic populations are to be found deeper than the reach of our cores. The constant increase in pore water alkalinity demonstrates active mineralization of OM in all cores (Figure 3B).

**Figure 5:**
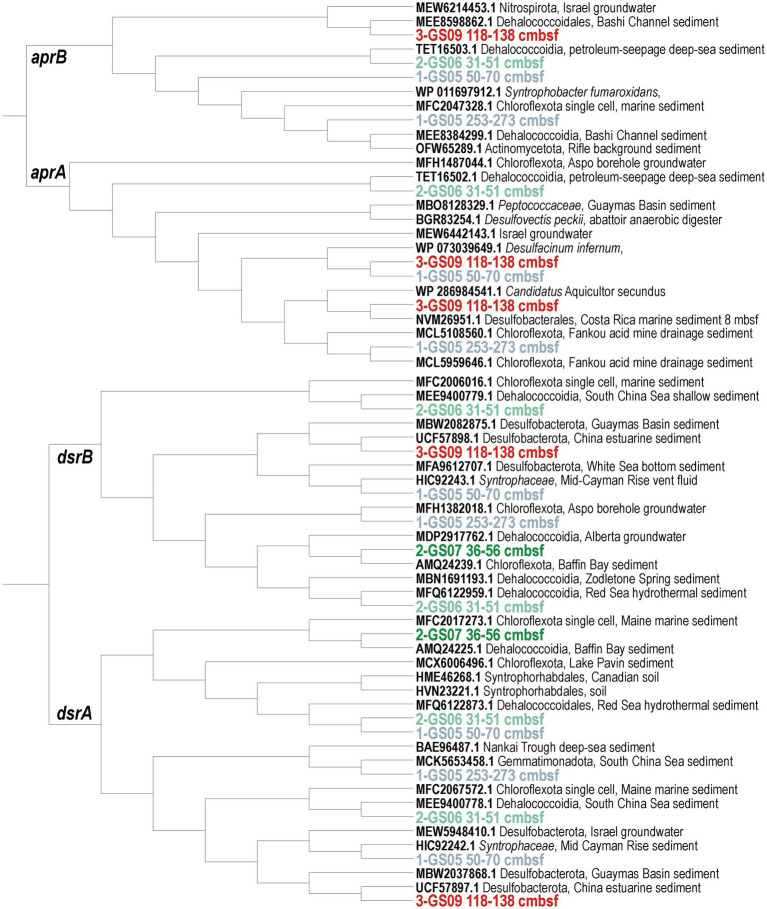
Phylogenetic trees of *aprAB* and *dsrAB* protein-encoding genes. PhyML maximum likelihood trees of open reading frames encoding conserved regions of the adenylyl-sulfate reductase subunit alpha (*aprA*) and beta (*aprB*); and dissimilatory sulfite reductase subunit alpha (*dsrA*) and beta (*dsrB*). Phylogenetic trees are based on 100 bootstrap replicates with BLOSUM62 as the evolutive model. Boldface type signifies sequence accession numbers to the NCBI database.

Considering sulfur cycling (Figure 3C), ORFs related to SO_4_^2−^ uptake and SO_3_^2−^ assimilation (*APS, APS-kn, Cys I*) and their respective transporters were found to be more abundant than ORFs encoding dissimilatory respiration of sulfur species (*apr*, *dsr*, *asr*, *psr*, *hyd*), dimethyl sulfide and sulfoxide (*dms*, *dmso*). Noteworthy is the increased relative abundance of ORFs encoding *sudA*. The convex-down curvature of pore water SO_4_^2−^ profiles argues for increased consumption between 1–2 mbsf (i.e., reactive layer). Phylogenetic analyses of protein conserved regions confirmed metabolic potential for dissimilatory sulfate reduction (*aprAB*, *dsrAB*) mostly among the class Dehalococcoidia at Site 1 and Site 2, and Desulfobacteria at Site 3 (Figure 5).

### 3.3 Diversity and abundance of *rpS3* gene sequences and metagenome-assembled genomes

**Figure 6:**
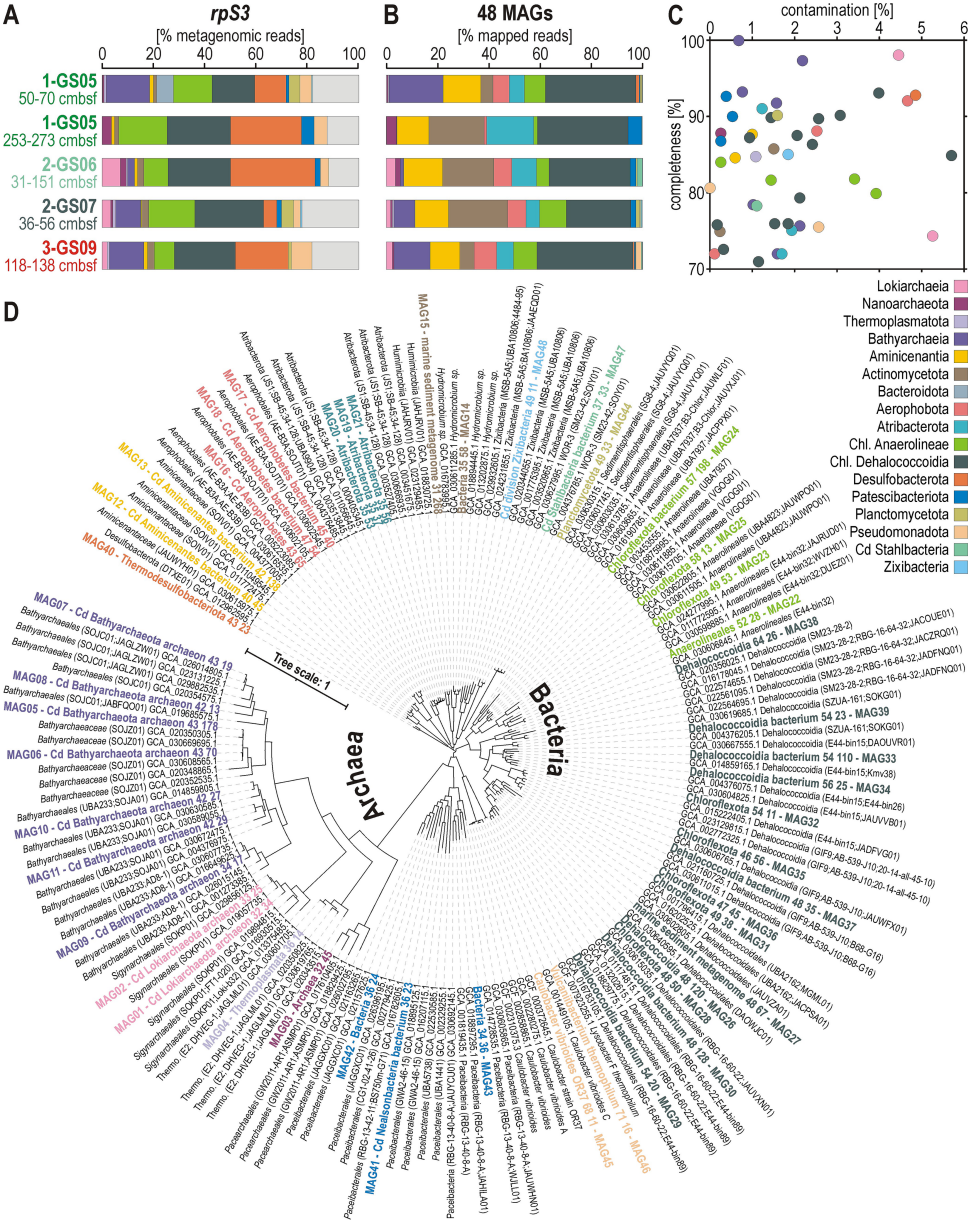
Taxonomy and relative abundances of *rpS3* gene and metagenome-assembled genomes. **(A)** Sample-level *rpS3* gene diversity and relative abundances at the phylum/class level. **(B)** Sample-level MAG relative abundances (at phylum/class level) based on mapped metagenomic reads normalized to each MAG respective genome size. **(C)** Degree of contamination (*X* axis) and completeness (*Y* axis) of the 48 MAGs obtained from hybrid assembly. **(D)** Phylogenetic tree based on 16 concatenated ribosomal proteins extracted from the 48 MAGs and their closely affiliated representative MAGs from the GTDB database with accession numbers.

The *rpS3*-based taxonomic diversity emphasizes the following phyla as the most abundant ones across all five metagenomes, namely Chloroflexota (class Dehalococcoidia, Anaerolineae), and Desulfobacterota (class Desulfobacteria, Desulfuromonadia) among Bacteria, and Thermoproteota (class Bathyarchaeia) and Asgardarchaeota (class Lokiarchaeia) among Archaea (Figure 6A). Phylogenetic analyses of *rpoD* (Bacteria) and *TFIIB* (Archaea) protein sequences extracted from contigs could confirm this distribution of taxonomic diversity (Supplementary Figures S4, S5).

About 20–30% of Illumina metagenomic reads could be assembled into contigs independently (Supplementary Table S2), mapped on MAGs (Supplementary Figure S6), and further improved through hybrid assembly with ONT long reads (Supplementary Table S3). Relative abundances of mapped short reads and *rpS3* show some variation, e.g., increased abundance of Acidobacteriota (class Aminicenentia), Actinomycetota and Atribacterota, but otherwise also document Chloroflexota and Bathyarchaeia as the main taxonomic clades (Figure 6B). A total of 48 MAGs with completeness ≥70% and contamination ≤10% were obtained (Figure 6C), with taxonomic assignments (Figure 6D) mostly among the phyla Chloroflexota (14 Dehalococcoidia, 4 Anaerolineae) and Thermoproteota (7 Bathyarcheaia), followed by Atribacterota (3), Aerophobia (3) and Patescibacteriota (3). Despite high relative abundance in *rpS3* genes (≤20%), we recovered only a single species-level MAG assigned to Desulfobacterota (Supplementary Table S4). Since gene-level analyses only require the recovery of scaffolds for prediction and annotation, it is possible to obtain more quantitative results than with high-quality MAGs.

### 3.4 Genetic content of metagenome-assembled genomes

**Figure 7:**
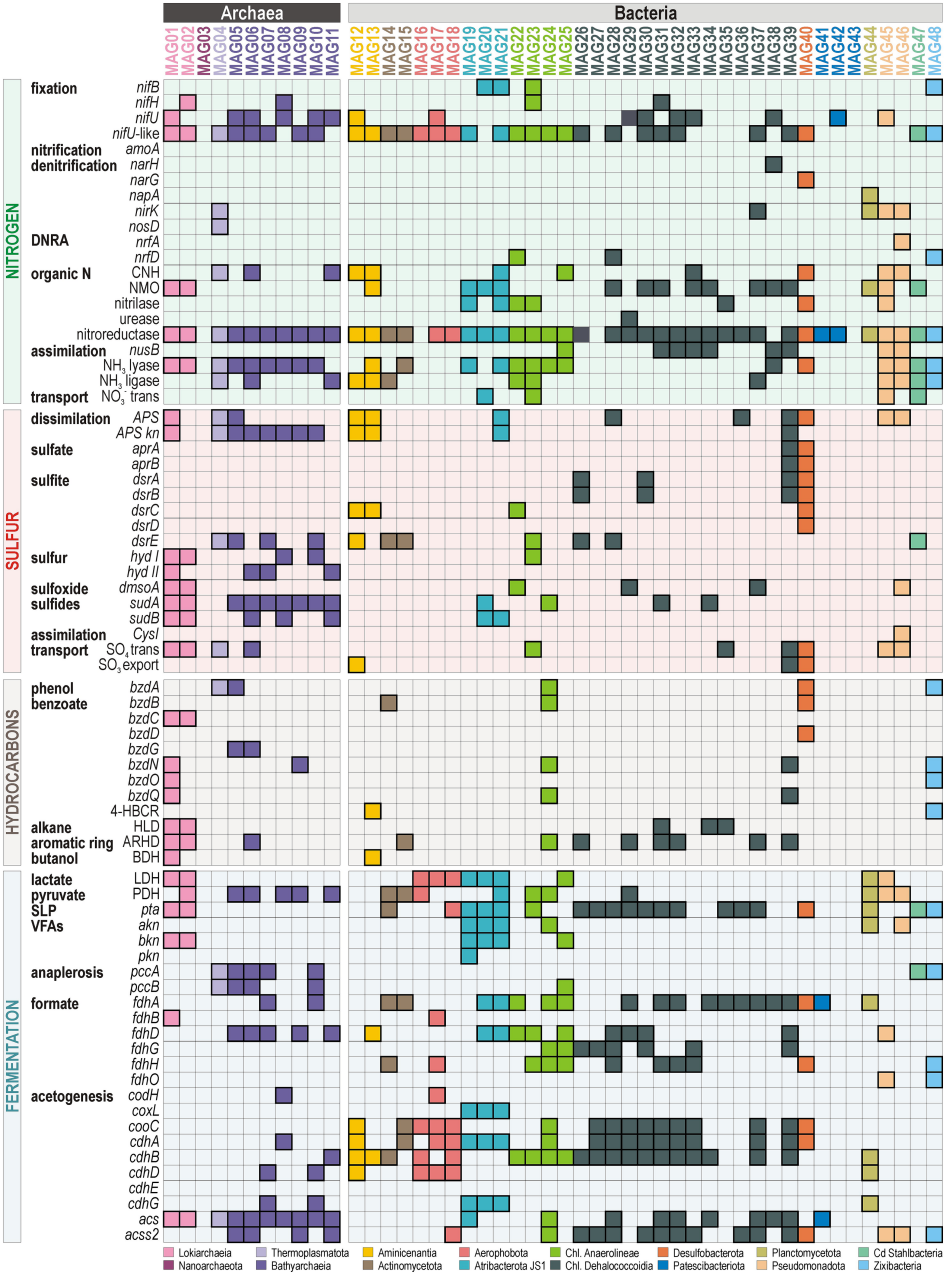
Presence/absence of functional marker genes related to biogeochemical nitrogen, sulfur, and carbon cycling. Presence/absence of functional marker genes related to nitrogen, sulfur, and carbon cycling in each of the 48 MAGs listed according to their taxonomic assignments. Enzymatic names and their corresponding gene abbreviations are listed in the supplement (Supplementary Table S1).

We queried the presence/absence of the same series of functional marker genes, as described above (Supplementary Table 1), across the predicted gene collection for the 48 MAGs retrieved (Figure 7). Metabolic potential for nitrogen fixation (*nifH*, *nifU*) is clearly present in MAGs assigned to Lokiarchaeia, Bathyarchaeia and Dehalococcoidia, otherwise sparsely identified in Aminicenentia, Aerophobotia and Alphaproteobacteria (*Caulobacter* sp.). Genes involved in denitrification *per se* were hardly identified, pointing mostly to MAGs of Pseudomonadota, Desulfobacterota and Planctomycetota, whereas DNRA-related marker genes (*nrfA*, *nrfD*) are found in Chloroflexota and Zixibacteria (Figure 7). Intracellular production of NO_2_^−^ and NO^−^ can be achieved via nitronate monooxygenase (*NMO*) and nitric oxide synthase (*NOS*), while metabolic potential for assimilatory nitrite reduction (*nirB*) is present in some MAGs assigned to Thermoplasmatota (DHVEG-1), Bathyarchaeia and Dehalococcoidia. By contrast, Atribacterota and Anaerolineae utilize and assimilate specific N-bearing C_1_ compounds (e.g., urea, nitrile). Organic N oxidation and NH_3_ assimilation are generally present in most MAGs (*CNH*, nitroreductase, NH_3_ ligase and lyase, *nusB*).

Marker genes for dissimilatory sulfate and sulfite reduction (*aprAB*, *dsrAB*) reveal specific MAGs assigned to clades of Dehalococcoidia and Desulfobacterota as sulfate-reducing bacteria (SRB). ORFs encoding genes predicted to function in the reduction of sulfur/polysulfide (*hyd I-II*) and/or ferredoxin (*sudAB*) are mostly found in Lokiarchaeia and Bathyarchaeia (Figure 7). In addition, Lokiarchaeia and Chloroflexota show metabolic capacity to reduce dimethyl sulfoxide (*dmsoA*). All related MAGs have metabolic potential for SO_4_^2−^ uptake and transport (*APS*, *APS kn*), with *dsrC-E* subunits that function as carrier in assimilation in sulfite reduction or sulfur oxidation (Ruiz-Blas et al., 2024).

Metabolic potential towards HC degradation via the benzoyl-CoA reductase pathway (*bzdA-O*) is mostly found in MAGs assigned to Lokiarchaeia, and to a lesser extent in Bathyarchaeia and Dehalococcoidia (Figure 7). Oxidation of HC aromatic rings (*ARHD*) and alkanes (*HLD*) is prominent in Dehalococcoidia and Lokiarchaeia. In terms of sediment carbon cycling, functional marker genes most often identified across MAGs are associated with fermentation (*LDH*, *PDH*) and (homo)acetogenesis (*fdhA-O*, *codH*, *cdhA-G*) with acetyl-CoA synthesis for energy production (*acs*) and biomass (*acss2*). In addition, anaplerotic CO_2_ fixation (*pccAB*) is mostly found in Bathyarchaeia, while SLP of VFAs (*akn*, *bkn*, *pkn*) is characteristic to MAGs of Atribacterota. Phosphotransacetylase (*pta*), which can also convert acetyl-CoA into acetate and biomass, is quite systematically present in MAGs of Chloroflexota.

Results of the FeGenie pipeline (Garber et al., 2020) on contigs show a high detection of metabolic potential for Fe acquisition and transport using siderophores, regulation and storage. In comparison, the metabolic potential related to Fe redox processes is minor, its detection being limited to Site 2 and Site 3 (Supplementary Figure S7). The same applies to archaeal and bacterial MAGs among which metabolic capacity for dissimilatory Fe reduction is phylogenetically restricted to two MAGs attributed to Desulfobacterota and Zixibacterota (Supplementary Figure S7). Yet, these results highlight the importance to acquire reactive Fe (via siderophores) which represents the limiting nutrient in deep waters off the Barents Sea (Rijkenberg et al., 2018).

## 4 Discussion

### 4.1 Metabolic potential for nitrogen cycling in oligotrophic sediments

During summer in the Barents Sea, phytoplankton consume and deplete NO_3_^−^ in surface waters, while bottom waters accumulate a certain amount (ca. 12 μM) due to primary OM breakdown and subsequent nitrification in the bottom water (Tuerena et al., 2021). Thus, denitrification can only represent a minor process in pelagic sediments compared to continental shelf sediments (Cheung et al., 2024). However, microaerobic conditions in the shallow subsurface would enable NO_3_^−^ to be regenerated through benthic NH_4_^+^ production from sediment OM, upward diffusion of pore water NH_4_^+^ and nitrification (Bonaglia et al., 2017). Pore water concentrations in NO_3_^−^/NO_2_^−^ were only measurable at Site 3 (10–30 μM), with geochemical gradients indicative of ongoing denitrification with slow NH_4_^+^ accumulation in pore water from 10 to 100 cmbsf (Figure 2C). The complete absence of pore water NO_3_^−^/NO_2_^−^ at Site 1 and Site 2 suggested prior consumption in the water column and dysoxic conditions near the SWI. In the seemingly near to complete absence of NO_3_^−^ in pore water, the curved NH₄^+^ profile indicated constant production via OM mineralization (i.e., ammonification) rather than DNRA (Bonaglia et al., 2017). Ammonification releases non-carbonate organic bases (e.g., NH_4_^+^, fulvic, humic acids) that also contribute to the increase in alkalinity observed (Lukawska-Matuszewska, 2016).

Overall, ORFs encoding genes with predicted activity in denitrification (i.e., *nar*, *nap*, *nir*, *nor*, *nos*) and DNRA (i.e., *nrf*) were few while metabolic potential for anammox (*hzo*) was even absent (Reyes et al., 2017). In sediments from Site 2 and Site 3, ORFs encoding genes for ammonia oxidation (*amoBC*) and denitrification (*narGH*, *napA*, *nirK*) were consistently detected. Metabolic potential for nitrifier denitrification (*amoBC*, *nirK*) in *Nitrosopumilus* sp. (Vuillemin, 2023) allows to deduce hypoxic conditions near the SWI at Site 2 (Figure 4). Further respiratory nitrate reduction involved taxa among the class Dehalococcoidia, Desulfobacteria and Aminicenentia. By contrast, the related predicted functions in MAGs did not reveal potential for nitrification, but only for respiratory nitrate/nitrite reduction by Planctomycetota, Pseudomonadota, Chloroflexota and Desulfobacterota (Figure 7).

Thus, pore water profiles (Figures 2, 3) and predicted functional marker genes together suggested ongoing nitrification–denitrification in sediments at Site 3 (i.e., pristine seabed) with gradual depletion of pore water electron acceptors (Figure 2C). In comparison at Site 2 (i.e., biogenic methane anomalies), nitrifier denitrification (i.e., NH_4_^+^ oxidation coupled to NO_2_^−^ reduction) and heterotrophic denitrification processes were inferred to take place in the water column followed by partial denitrification in the sediment under limited pore water nitrate. Iron acquisition via siderophores and redox processes potentially coupled to nitrate reduction (Benz et al., 1998) were only detected at Site 2 and Site 3 (Supplementary Figure S7). Geochemical conditions thus appeared to be more reducing at Site 1. This site is characterized by thermogenic methane anomalies, which expectedly reflect the higher availability of favorable organic substrates (Scott et al., 2014). Higher substrate availability was equally reflected in slightly higher cell densities, CH_4_ and CO_2_ concentrations (Figure 2B), and steeper pore water geochemical profiles (Figure 3). However, no clear differences were observed in the *rpS3* taxonomic diversity across all three sites (Figure 6), which may point to metabolic versatility within taxonomic clades.

### 4.2 Metabolic versatility of functional guilds across geochemical niches

Downcore concentration profiles for pore water sulfate, alkalinity and ammonium (Figures 3A–C) appeared consistent with microbial sulfate reduction coupled to OM remineralization being the quantitatively dominant process (Schnabel et al., 2025a). Organoclastic sulfate reduction consumes pore water sulfate (SO_4_^2−^) and generates bicarbonate (HCO₃^−^) as a major component of alkalinity. The relative abundance of *rpS3* genes in the sediment interval exhibiting the strongest sulfate drawdown highlighted the phyla Desulfobacterota and Chloroflexota (Figure 6A). These two phyla commonly include SRB (Wasmund et al., 2017; Vuillemin et al., 2022). Phylogenetic trees of the respective marker proteins (*aprAB*, *dsrAB*) demonstrated predicted potential for dissimilatory sulfate reduction (Figure 5) among some Chloroflexota (class Dehalococcoidia) and Desulfobacterota (class Syntrophia, Syntrophobacteria, Desulfobacteria), which was further confirmed by their related MAGs (Figure 7). Their metagenomic analysis provided evidence for metabolic versatility in the use of nitrate, sulfate and iron as electron acceptors (Figures 4, 5; Supplementary Figure S7). Carbon assimilation could proceed through the degradation of aromatic HCs via the benzoyl-CoA pathway (Supplementary Figure S3) and oxidation of VFAs and molecular hydrogen as electron donors in (homo)acetogenic fermentation (Figures 3B, 7). Consortia of SRB are also known to potentially fix N₂ under anoxic conditions (Bertics et al., 2013; Dekas et al., 2018). This was partially confirmed by the genetic content of these MAGs, with ORFs encoding *nifH* and *nifU* proteins (Figure 7; Supplementary Figure S2). Thus, the key SRB in Barents Sea sediments exhibit an adaptive metabolism predicted to use the most favorable electron donors and acceptors available in their direct surroundings.

In addition, (homo)acetogenic fermenters among the classes Dehalococcoidia, Lokiarchaeia and Bathyarchaeia also display a wide metabolic versatility. These classes not only utilize diverse organic carbon compounds (Figures 3B, 7), but also interact with various sulfur and nitrogen redox intermediates. For instance, by-products of sulfate reduction (i.e., dimethyl sulfoxide, sulfur) could be reduced or consumed by bacterial and archaeal clades among Chloroflexota (i.e., *dmsoA*, *dsrE*), Atribacterota (i.e., *APS*, *APS-kn*, *sudAB*), Lokiarchaeia (i.e., *APS*, *APS-kn*, *dmsoA*, *hyd I-II*, *sudAB*) and Bathyarchaeia (i.e., *hyd I-II*, *sudAB*, *dsrE*). All these clades are known to be fermenters capable of conserving redox energy via an (homo)acetogenic WLP coupled to different electron donors and acceptors (Ruiz-Blas et al., 2024; Vuillemin et al., 2020a, 2020b, 2024). The corresponding MAGs showed consistent metabolic potential towards assimilation of simple organic nitrogen compounds (*CNH*, *NMO*, nitrilase, urease) and diazotrophy with ORFs encoding *nifH* and *nifU* proteins (Figure 7; Supplementary Figure S2). Because nitrogenase requires 8 electrons and 16 ATP per N_2_ fixed (Martínez-Espinosa et al., 2011), diazotrophic fermenters rather utilize different enzymes to generate low potential reducing equivalents via electron bifurcation, e.g., ferredoxin, flavodoxin (Alleman and Peters, 2023). Although nutrient limitation usually promotes N_2_ fixation (Dynarski and Houlton, 2018), less energy-demanding production of ammonia (Herrero et al., 2019) can be achieved by converting diverse sources of organic N (e.g., nitroreductase) and assimilated (e.g., ammonia ligase, lyase). However, under limited OM sedimentation rates, sulfur and sulfate reducers may have to perform nitrogen fixation in the sediment concurrently with denitrification (Fulweiler et al., 2013). Noteworthy, clades of Dehalococcoidia exhibited metabolic capacity for respiratory nitrate and sulfate reduction with nitrogen fixation, and HC degradation coupled with energy conservation in the WLP (Figure 7).

### 4.3 Hydrocarbon degradation and cross-feeding among versatile acetogens

Genetic content of the MAGs emphasized metabolic flexibility of specific (homo)acetogens in resource utilization, and confirmed the widespread nature of N_2_ fixation in anoxic sedimentary environments (Dekas et al., 2018), especially under reduced organic carbon loading. However, HC seepage from deeply buried reservoirs may supply additional electron donors from below and stimulate *in situ* microbial activity compared to pristine sediments (Schnabel et al., 2025a).

Specific microbial clades that potentially play a key role in anaerobic degradation of HCs (i.e., alkanes, aromatic rings) included a majority of (homo)acetogenic fermenters (e.g., Lokiarchaeia, Bathyarchaeia, Aminicenentia, Anaerolineae, Dehalococcoidia) (Figure 7; Supplementary Figure S3). These classes already exhibited remarkable metabolic versatility in dissimilatory nitrogen and sulfur metabolism along with N_2_ fixation (Deb and Das, 2022; Dong et al., 2022). In addition, MAGs of Actinobacteriota, Desulfobacterota, and Zixibacteria included specific ORFs encoding a partial reductive pathway for benzoate degradation (Figure 7). These microorganisms can apparently contribute in concert to the degradation of simple aromatic HCs, thereby producing fermentative metabolites (e.g., VFAs) to support energy needs of various fermenters able to perform SLP (e.g., Actinobacteriota, Atribacterota, Aerophobota, Planctomycetota, Dehalococcoidia). This indicates that HC degradation may significantly alter biogeochemical cycling processes by either promoting cross-feeding interactions on metabolites or exerting selective pressure on canonical denitrifiers and sulfate reducers due to the use of electron acceptors by versatile fermenters (Hanke et al., 2016). In the absence of HC-derived electron donors, the same (homo)acetogens could perform fermentative breakdown of proteins instead and use organic residues that canonical denitrifiers and SRB cannot assimilate, similar to deep biosphere environments facing substrate limitation (Liu et al., 2022).

Altogether, our results combining pore water geochemistry and metagenomics highly suggest that (homo)acetogenic fermentation, when fueled by diffusive HC seepage, supports cross-feeding interactions. In particular, those associated with dissimilatory sulfate reduction and recycling of its by-products appear to be potentially combined with cryptic nitrate reduction and N_2_ fixation. In pristine seabed not exposed to seepage (i.e., Site 3), NO_3_^−^ and NH_4_^+^ profiles were consistent with denitrification down to 1 mbsf. Under minor HC seepage, the available organic carbon sources and electron donors led to very different pore water profiles, with little impact on community diversity. This could be explained by metabolic versatility across functional guilds (Dal Bello et al., 2021), which seemed to further limit geochemical differentiation of sedimentary niches. This favored polyvalent (homo)acetogens (i.e., Lokiarchaeia, Bathyarchaeia, Dehalococcoidia) at the expense of canonical denitrifiers and sulfate reducers. Altogether, these diazotrophic (homo)acetogens, which are slow-growing but ubiquitous in the marine subseafloor, appeared to support complex interactions that contributed to balancing biogeochemical cycles in sedimentary environments impacted by minor HC seepage.

## 5 Conclusion

Our study shows how minor HC seepage can enhance microbial redox processes in oligotrophic sediments of the Barents Sea. By combining pore water geochemistry and metagenomics, we demonstrate that, when fueled simple HCs through diffusive seepage, metabolic versatility by specific (homo)acetogens fosters cross-feeding interactions, particularly those associated with dissimilatory sulfur metabolism and fermentation of intermediate metabolites. Geochemical profiles showed that denitrification could only proceed in pristine organic-lean sediments, whereas additional organic carbon substrates (i.e., hydrocarbons) apparently promoted faster consumption of electron acceptors by versatile (homo)acetogenic fermenters. Among those, clades of Dehalococcoidia were capable of nitrate and sulfate reduction coupled with HC degradation and cryptic N_2_ fixation, thereby outcompeting canonical denitrifiers and sulfate reducers. In the absence of HC-derived electron donors, Dehalococcoidia, Atribacteria, Lokiarchaeia and Bathyarchaeia are capable of (homo)acetogenic fermentation of organic residues and energy conservation in the WLP. Thus, ubiquitous slow-growing (homo)acetogens appear to support complex cross-feeding interactions inherent to their metabolic versatility independent of geochemical niches, thereby contributing to conservative biogeochemical cycles in sedimentary environments impacted by minor HC seepage.

## Data Availability

The raw metagenome sequencing data are publicly available on the NCBI database (www.ncbi.nlm.nih.gov) via the project accession number PRJNA1175466. The geochemical data are publicly available on the PANGAEA^®^ Data Publisher as dataset #974346 (https://doi.pangaea.de/10.1594/PANGAEA.974421). All scripts and instructions regarding how to conduct the BLASTp workflow for functional annotations and the related aggregated genome database are available on the MetaProt page (https://data.ub.uni-muenchen.de/183/).

## Footnotes

1
https://github.com/rrwick/Filtlong

2
https://github.com/0x4D31/galah

## Group members of the PROSPECTOMICS Consortium

The principal investigators of the project are: Jens Kallmeyer, GFZ Helmholtz Centre for Geosciences, Section Geomicrobiology, Telegrafenberg, 14,473 Potsdam, Germany; Paul Wilmes, Luxembourg Centre for Systems Biomedicine, University of Luxembourg, Esch-sur-Alzette; Alexander J. Probst, Environmental Metagenomics, Research Center One Health Ruhr of the University Alliance Ruhr, Faculty of Chemistry, University of Duisburg-Essen, Essen, Germany; Dörte Becher, Department of Microbial Proteomics, University of Greifswald, Greifswald, Germany; Thomas Rattei, Computational Systems Biology, Centre for Microbiology and Environmental Systems Science, University of Vienna, Vienna, Austria; and Rolando di Primio, Aker BP ASA, Sandvika, Viken, Norway. Section Organic Geochemistry, Telegrafenberg, 14,473 Potsdam, Germany. Their project managers are Aurèle Vuillemin, GFZ Helmholtz Centre for Geosciences, Section Geomicrobiology, Telegrafenberg, 14,473 Potsdam, Germany; Cédric C. Laczny, Luxembourg Centre for Systems Biomedicine, University of Luxembourg, Esch-sur-Alzette; André R. Soares, Environmental Metagenomics, Research Center One Health Ruhr of the University Alliance Ruhr, Faculty of Chemistry, University of Duisburg-Essen, Essen, Germany; and Anke Trautwein-Schult, Department of Microbial Proteomics, University of Greifswald, Greifswald, Germany. Scientists and technical staff include Ellen Schnabel, GFZ Helmholtz Centre for Geosciences, Section Geomicrobiology, Telegrafenberg, 14,473 Potsdam, Germany; Kai Mangelsdorf, GFZ Helmholtz Centre for Geosciences, Section Organic Geochemistry, Telegrafenberg, 14,473 Potsdam, Germany. Steffen Okolski, GFZ Helmholtz Centre for Geosciences, Section Geomicrobiology, Telegrafenberg, 14,473 Potsdam, Germany; J. Axel Kitte, GFZ Helmholtz Centre for Geosciences, Section Geomicrobiology, Telegrafenberg, 14,473 Potsdam, Germany; Benoît J. Kunath, Luxembourg Centre for Systems Biomedicine, University of Luxembourg, Esch-sur-Alzette; Oskar Hickl, Luxembourg Centre for Systems Biomedicine, University of Luxembourg, Esch-sur-Alzette; Tuesday Lowndes, Luxembourg Centre for Systems Biomedicine, University of Luxembourg, Esch-sur-Alzette; Zainab Zafar, Luxembourg Centre for Systems Biomedicine, University of Luxembourg, Esch-sur-Alzette; Sarah Esser, Environmental Metagenomics, Research Center One Health Ruhr of the University Alliance Ruhr, Faculty of Chemistry, University of Duisburg-Essen, Essen, Germany; Anne Ostrzinski, Department of Microbial Proteomics, University of Greifswald, Greifswald, Germany; Sebastian Grund, Department of Microbial Proteomics, University of Greifswald, Greifswald, Germany; and Alexander Pfundner, Computational Systems Biology, Centre for Microbiology and Environmental Systems Science, University of Vienna, Vienna, Austria.
