# Supplementary material for: Geochemical variability and microbial metabolic functions in oligotrophic sediments exposed to minor seepage
Source: Front Microbiol. 2026 Jan 21;16:1720187. doi: 10.3389/fmicb.2025.1720187 (PMC12869995; doi:10.3389/fmicb.2025.1720187)
Supplement: Supplementary file 1 [file Data_Sheet_1.PDF]

Supplementary Material

Geochemical variability and microbial metabolic functions in oligotrophic sediments exposed to minor seepage

Ellen Schnabel, Aurèle Vuillemin*, Sarah Esser, Lea Griesdorn, André R. Soares, Pål Tore Mørkved, Steffen L. Jørgensen, Alexander J. Probst, Jens Kallmeyer and the PROSPECTOMICS Consortium

List of content

Supplementary Figures

- **Supplementary Figure S1.** Regional overview, bathymetry and HC manifestations of the sampling area
- **Supplementary Figure S2.** Phylogenetic tree of nitrogen fixation protein sequences (*nifB-X*)
- **Supplementary Figure S3.** Phylogenetic tree of benzoyl-CoA reductase protein sequences (*bzdNOQ*)
- **Supplementary Figure S4.** Phylogenetic tree of protein sequences annotated as *rpoD* genes (Bacteria)
- **Supplementary Figure S5.** Phylogenetic tree of protein sequences annotated as *TFIIB* genes (Archaea)
- **Supplementary Figure S6.** Total Illumina metagenomic reads mapped onto hybrid MAGs
- **Supplementary Figure S7.** Results of the FeGenie pipeline for contigs and hybrid MAGs

Supplementary Tables

- **Supplementary Table S1.** List of enzymatic names and abbreviations for functional marker genes involved in sediment biogeochemical cycles
- **Supplementary Table S2.** Metadata on *de novo* assembly of Illumina metagenomic reads into contigs
- **Supplementary Table S3.** Metadata on hybrid assembly of Illumina and Oxford Nanopore Technology (ONT) metagenomic reads into scaffolds
- **Supplementary Table S4.** Metadata on hybrid assembly of metagenome-assembled genomes (MAGs)

Supplementary Data

- **Supplementary Data.** Sequencing data (*rpS3*, BlastP results) and metadata provided as a separate excel sheet (Supplementary Data.xlsx)

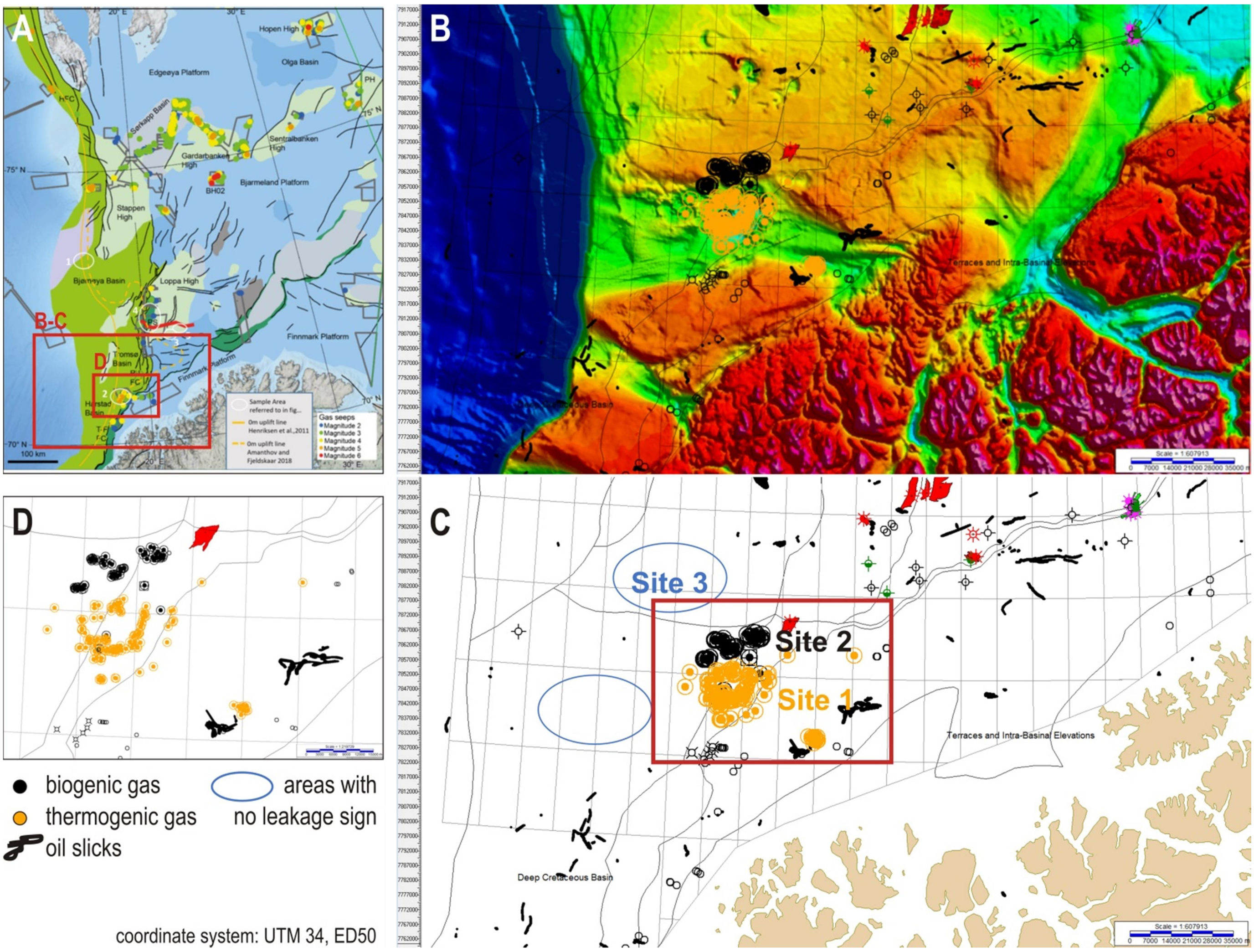

Supplementary Figure S1. Regional overview of the seep area, with successive close-ups depicting the bathymetric topography and hydrocarbon manifestations at the three sampling locations. (A) Regional overview of the main geological elements highlighting the seep area of this study (square); **(B)** Close-up to the seep area (Håkjerringsdjupet) with bathymetric topography of the seafloor. Circles signify the occurrence of natural thermogenic (orange) and biogenic (black) gas seeps. **(C-D)** Hydrocarbon manifestations include oil slicks, gas seeps and pockmarks, whereas blue circles mark reference areas with no signs of seepage. Based on these manifestations, selective sampling sites were located for gravity coring operations, i.e. Site 1 (thermogenic methane), Site 2 (biogenic methane), and Site 3 (pristine seabed). Maps are modified from (Rise et al., 2015; Hansen, 2017; Matapour et al., 2018).

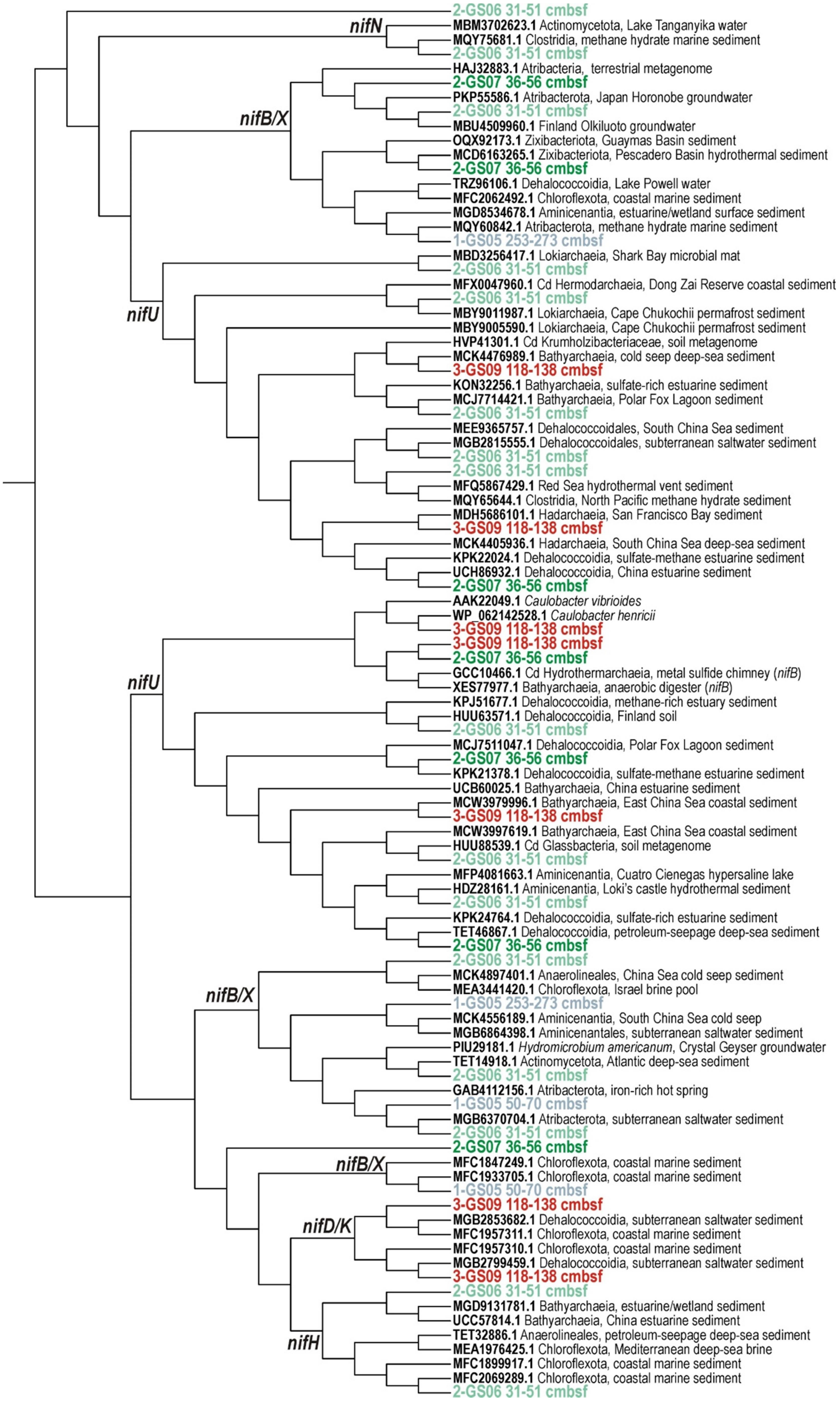

Supplementary Figure S2. Phylogenetic tree of protein-encoding genes involved in nitrogen fixation. PhyML maximum likelihood tree of open reading frames encoding conserved regions of nitrogen fixation proteins, i.e. Fe-Mo cofactor synthesis (*nifB-X*), dinitrogenase reductase (*nifH*), and Fe-S cluster assembly (*nifU*). The phylogenetic tree is based on 100 bootstrap replicates with BLOSUM62 as the evolutive model. Boldface type signifies sequence accession numbers to the NCBI database.

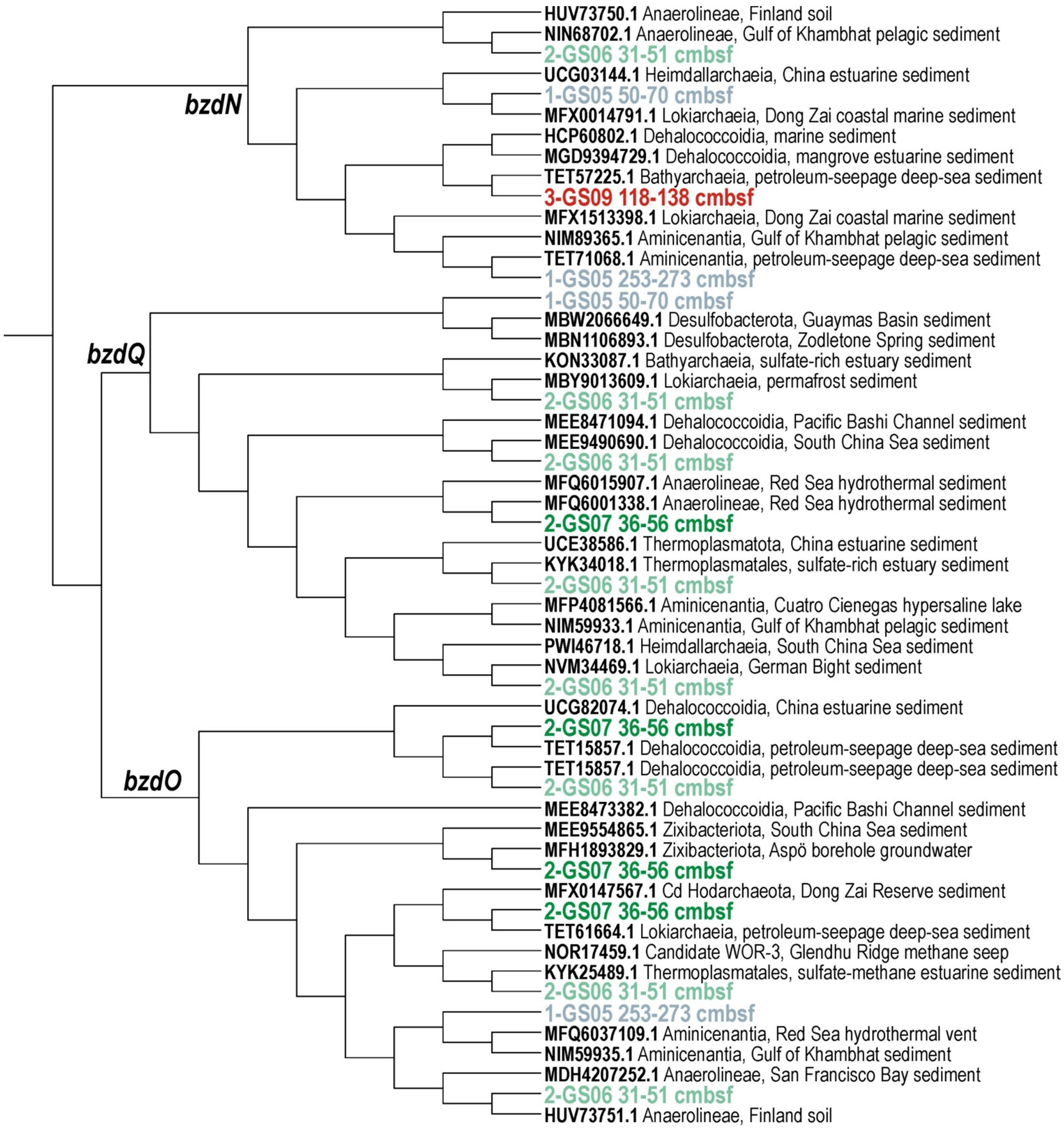

Supplementary Figure S3. Phylogenetic tree of benzoyl-CoA reductase protein-encoding genes. PhyML maximum likelihood trees of open reading frames encoding conserved regions of the benzoyl-CoA reductase N subunit (*bzdN*), O subunit (*bzdO*) and Q subunit (bzdQ). The phylogenetic tree is based on 100 bootstrap replicates with BLOSUM62 as the evolutive model. Boldface type signifies sequence accession numbers to the NCBI database.

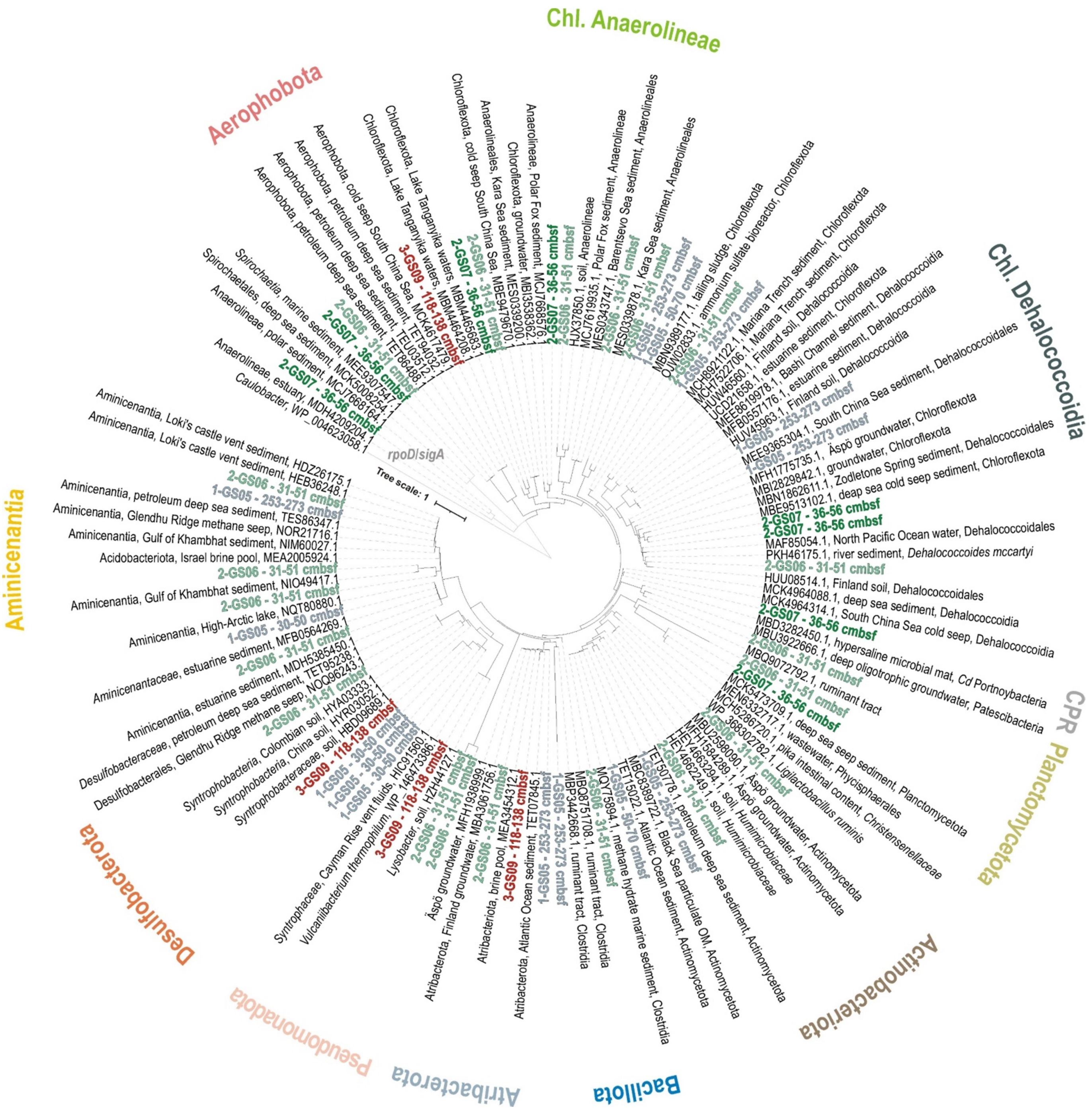

Supplementary Figure S4. Phylogenetic tree of protein sequences annotated as rpoD genes. PhyML Blosum62 phylogenetic tree of open-reading frames (ORFs) for conserved regions (167 amino acids) encoding the RNA polymerase sigma 70 factor (*RpoD*) extracted from bacterial contigs. The tree is based on 100 replicates, boldface types signify sequence accession numbers to the NCBI database.

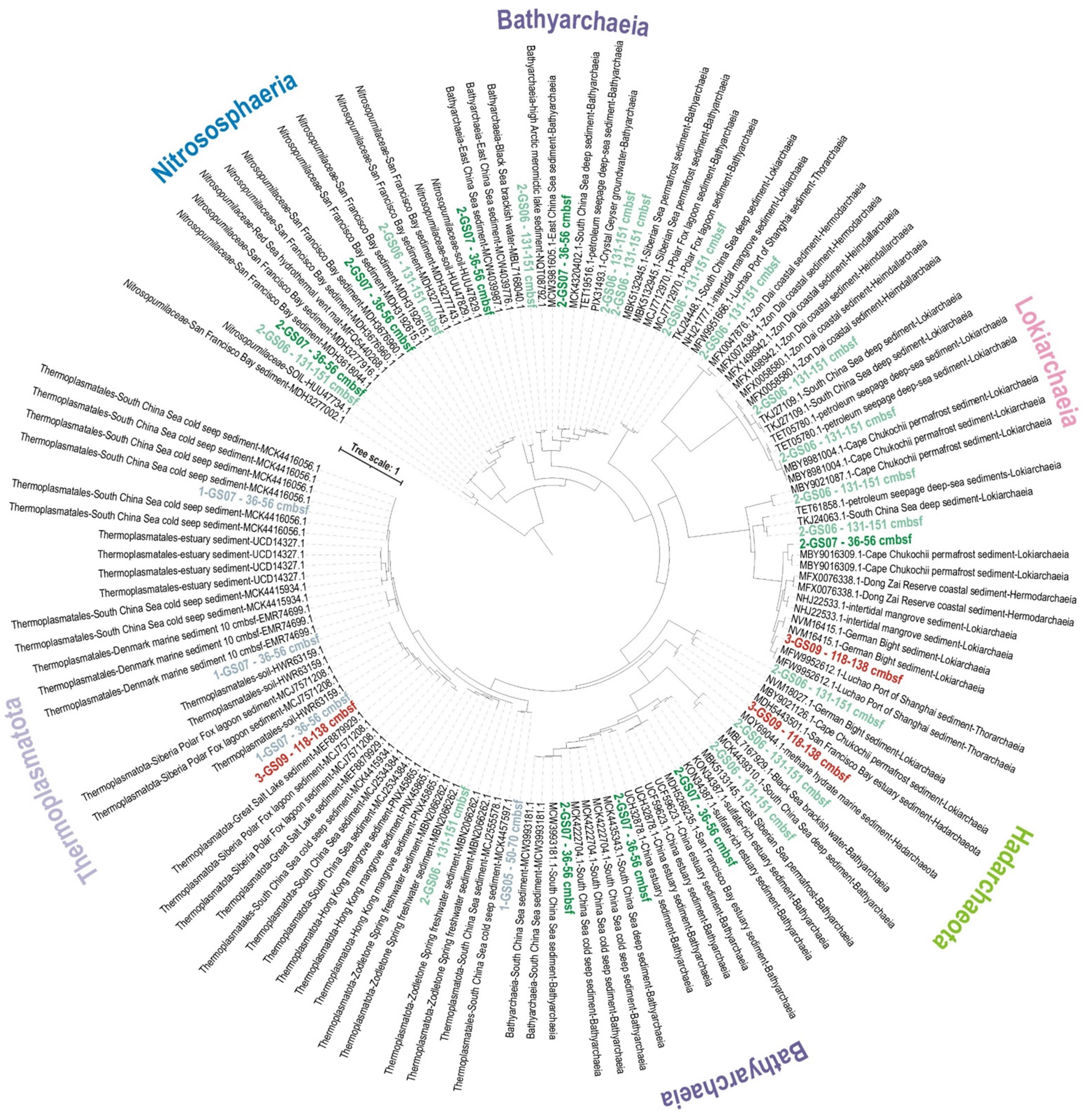

Supplementary Figure S5. Phylogenetic tree of protein sequences annotated as TFIIB genes. PhyML Blosum62 phylogenetic tree of open-reading frames (ORFs) for conserved regions (155 amino acids) encoding the transcription initiation factor IIB (*TFIIB*) extracted from archaeal contigs. The tree is based on 100 replicates, boldface types signify sequence accession numbers to the NCBI database.

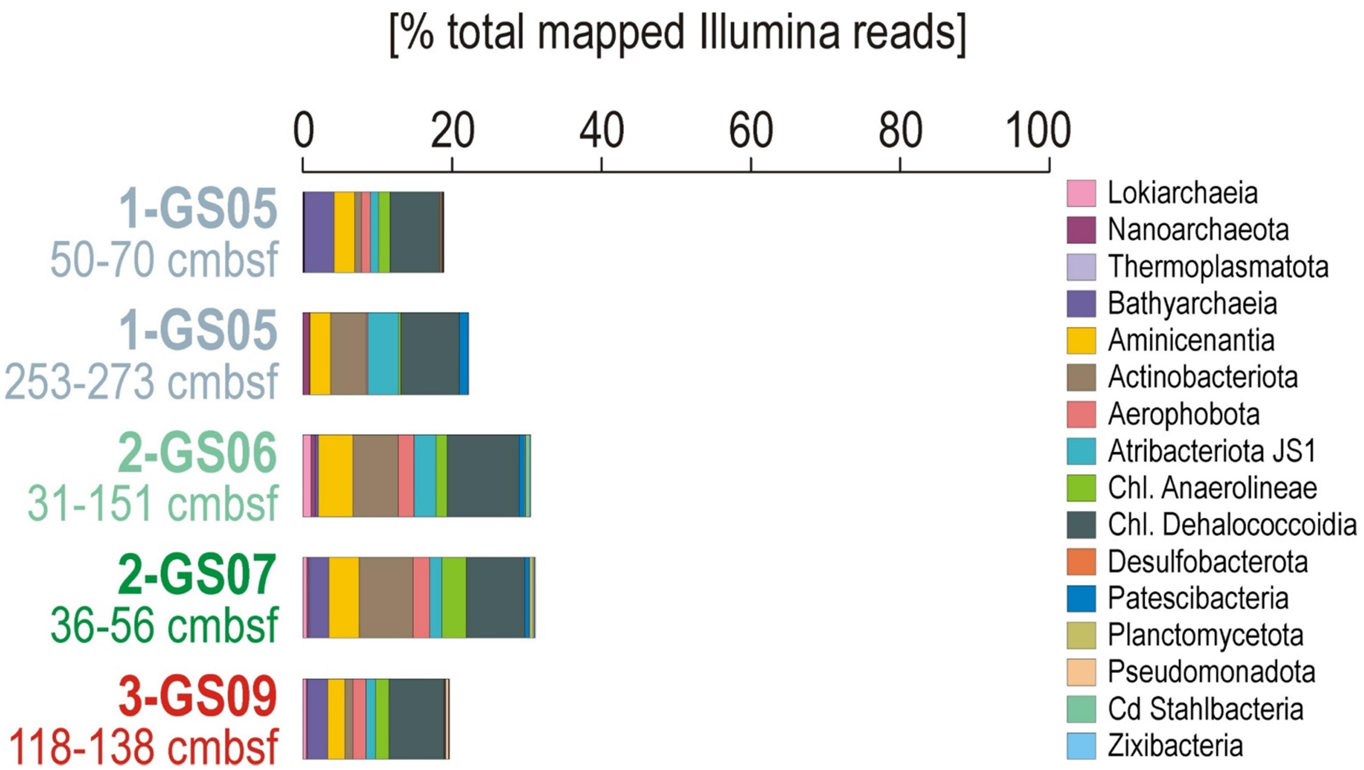

Supplementary Figure S6. Total Illumina metagenomic reads mapped onto MAGs. Normalized relative abundances [%] of total Illumina metagenomic reads mapped onto the 48 MAGs (X axis) obtained for each metagenome (Y axis) color-coded according to their taxonomic assignment at the phylum/class level.

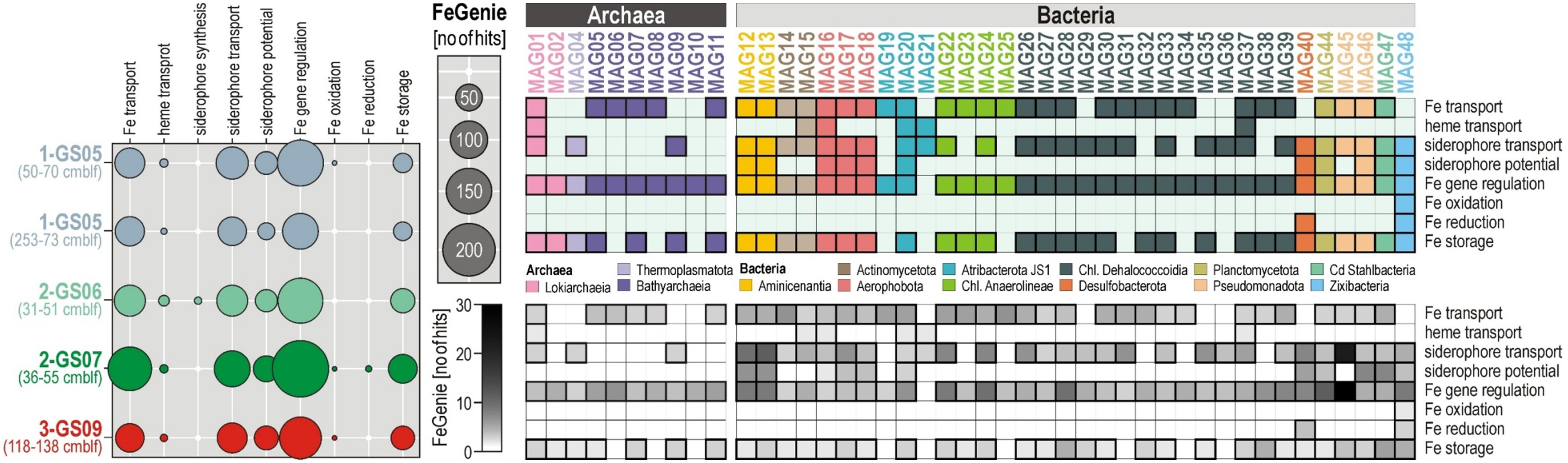

Supplementary Figure S7. (**Left**) Bubble plot of metabolic potential associated with iron processes in contigs assembled for the 5 Illumina short-read metagenomes. (**Right**) Heatmap detailing the presence/absence and counts of metabolic potential associated with iron processes for 44 MAGs. The 4 MAGs that had no hit to FeGenie clusters are not displayed (i.e. 1 Nanoarchaeota, 3 Patescibacteria).

Supplementary Table S1. List of enzymatic names and abbreviations for functional marker genes involved in sediment biogeochemical cycles.

Processes and pathways	Enzymes	Gene abbreviations
Taxonomy	RNA polymerase sigma 70 factor (Bacteria)	*rpoD*
	transcription initiation factor IIB (Archaea)	*TFIIB*
Nitrogen cycling<br>- fixation<br>- nitrification<br>- denitrification<br>- DNRA<br>- organic N sources<br>- assimilation	nitrogen fixation (diazotrophy)	*nifB-X*
	ammonia monooxygenase (nitrification)	*amoA-C*
	hydrazine oxidoreductase or dehydrogenase (anammox)	*hzo*
	respiratory nitrate reductase (denitrification)	*narG-H*
	periplasmic nitrate reductase	*napA-H*
	respiratory nitrite reductase	*nirK-S*
	anaerobic nitric oxide reductase (flavorubredoxin)	*norA-Q*
	nitrous oxidase accessory protein	*nos*
	ammonia-forming cytochrome c nitrite reductase (DNRA)	*nrfD*
	nitrogen fixation proteins (Fe-S cluster protein assembly)	*nifU-like*
	carbon-nitrogen hydrolase	*CNH*
	nitric oxide synthase (NO-producing)	*NOS*
	nitronate monooxygenase (NO_2^--producing)	*NMO*
	NAD(P)H-nitrite reductase (NO_2^--producing)	*nas*
	assimilatory nitrate reductase (NAD(P)H)	*nusB*
	nitrogen regulatory protein P-II	P_{II} sensor
Benzoyl-CoA pathway and hydrocarbon degradation	4-hydroxybenzoyl-CoA reductase	*HBCR*
	benzoyl-CoA reductase	*bzdA-Q*
	haloalkane dehydrogenase	*HLD*
	aromatic ring hydroxylase	*ARHD*
	aromatic ring-opening dioxygenase (catalytic subunit ligB)	*DODA*
	NADH-dependent butanol dehydrogenase	*BDH*
	ethylbenzene dehydrogenase	*EBDH*
Substrate-level phosphorylation (SLP) of volatile fatty acids (VFAs)	phosphotransacetylase	*pta*
	acetate kinase	*akn*
	butyrate kinase	*bkn*
	propionate kinase	*pkn*
Fermentation	lactate dehydrogenase	*LDH*
	pyruvate dehydrogenase	*pdhAB*
Anaplerosis	pyruvate carboxylase	*pccAB*
Acetogenesis	formate dehydrogenase	*fdhA-G*
	carbon monoxide dehydrogenase	*codh*
	aerobic carbon monoxide dehydrogenase (small/large)	*coxL-S*
	carbon monoxide dehydrogenase (nickel insertion)	*cooC*
	acetyl-coenzyme A synthase	*cdhA-G*
	acetyl-coenzyme A synthetase	*acs*
	acetyl-coenzyme A acetate ligase	*acss2*
Methanogenesis	acetyl-coenzyme M reductase (alpha subunit)	*mcrA*

Dissimatory sulfur metabolism and assimilation	adenylylsulfate transferase	*APS*
	adenylylsulfate kinase	*APS-kn*
	adenylylsulfate reductase (alpha and beta subunits)	*aprAB*
	dissimilatory sulfite reductase	*dsrA-K*
	polysulfide reductase	*psrC*
	sulfhydrogenase (I and II)	*hydA-G*
	dimethyl sulfoxide reductase	*dmsoAB*
	sulfide dehydrogenase (ferredoxin-dependent)	*sudAB*
	dimethyl sulfide dehydrogenase	*dmsAB*
	ferredoxin-dependent sulfite reductase	*Cys I*

Supplementary Table S2. Metadata on *de novo* assembly of Illumina metagenomic reads into contigs for the 5 samples whose libraries were successfully sequenced.

Illumina only	GS05 (50-70 cmbsf)	GS05 (253-273 cmbsf)	GS06 (30-50 cmbsf)	GS07 (36-55 cmbsf)	GS09 (118-138 cmbsf)
**Number of assembled reads**	11,568,654	9,252,946	14,726,938	24,539,528	8,563,686
**Assembled reads [%]**	19.12	22.45	30.42	31.31	19.64
**Number of scaffolds**	20,610	11,513	23,293	34,490	20,366
**Number of contigs**	25,868	14,299	30,381	41,237	26,034
**Contig length [bps]**	58,985,524	38,408,800	76,857,281	106,565,475	59,082,845
**Contig N50**	4,607	2,082	4,849	6,474	4,870
**Contig L50**	2,866	3,754	3,500	3,404	2,888
**Number of predicted genes**	74,303	45,302	91,346	125,855	72,729
**Number of predicted ORFs**	41,269	30,311	47,519	78,285	54,623

Supplementary Table S3. Metadata on hybrid assembly of Illumina and Oxford Nanopore Technology (ONT) metagenomic reads into scaffolds for the 5 samples whose libraries were successfully sequenced.

Oxford Nanopore Technology (ONT)	GS05 (50-70 cmbsf)	GS05 (253-273 cmbsf)	GS06 (30-50 cmbsf)	GS07 (36-55 cmbsf)	GS09 (118-138 cmbsf)
**Number of ONT base pairs (Gbps)**	0.29	0.30	0.27	0.14	0.25
**Read mean length [bps]**	3,129.90	3,258.49	3,061.20	3,082.81	3,221.16
**Number of Illumina base pairs (Gbps)**	11.39	8.15	9.61	12.90	8.30
**Number of predicted genes (hybrid)**	96,538	58,425	108,556	153,847	100,100
**Number of predicted ORFs (hybrid)**	60,968	36,171	70,210	88,720	63,531

Supplementary Table S4. Metadata on hybrid assembly of the 48 metagenome-assembled genomes (MAGs) obtained from the 5 metagenomic libraries successfully sequenced with Illumina and Oxford Nanopore Technologies.

MAG ID	MAG taxonomy (GTDB)	Completeness [%]	Contamination [%]	Genome size [bps]	ORF hits MetaCerberus	ORF hits MetaProt
MAG01	d_Archaea; p_Asgardarchaeota; c_Lokiarchaeia; o_Sigynarchaeales; f_SOKP01; g_SOKP01	98.11	4.45	3,636,222	2,561	1,954
MAG02	d_Archaea; p_Asgardarchaeota; c_Lokiarchaeia; o_Sigynarchaeales; f_SOKP01; g_SOKP01; s_SOKP01 sp029856125	74.39	5.25	2,648,021	2,214	1,729
MAG03	d_Archaea; p_Nanoarchaeota; c_Nanoarchaeia; o_Pacearchaeales; f_GW2011-AR1; g_ASMP01	87.8	0.27	561,171	180	366
MAG04	d_Archaea; p_Thermoplasmatota; c_E2; o_DHVEG-1; f_DHVEG-1; g_JAGLML01; s_JAGLML01 sp030601705	84.76	1.09	1,845,693	1,076	1,334
MAG05	d_Archaea; p_Thermoproteota; c_Bathyarchaeia; o_Bathyarchaeales; f_Bathyarchaeaceae; g_SOJZ01	93.21	0.77	1,654,339	1,026	1,498
MAG06	d_Archaea; p_Thermoproteota; c_Bathyarchaeia; o_Bathyarchaeales; f_Bathyarchaeaceae; g_SOJZ01	100	0.68	1,946,634	1,315	1,190
MAG07	d_Archaea; p_Thermoproteota; c_Bathyarchaeia; o_Bathyarchaeales; f_SOJC01; g_JAGLZW01; s_JAGLZW01 sp026014805	91.79	1.57	1,631,481	1,161	1,148
MAG08	d_Archaea; p_Thermoproteota; c_Bathyarchaeia; o_Bathyarchaeales; f_SOJC01; g_JAUWDF01	72.06	1.59	1,123,924	914	1,318
MAG09	d_Archaea; p_Thermoproteota; c_Bathyarchaeia; o_Bathyarchaeales; f_UBA233; g_AD8-1	78.47	1.02	1,454,733	1,005	1,246
MAG10	d_Archaea; p_Thermoproteota; c_Bathyarchaeia; o_Bathyarchaeales; f_UBA233; g_SOJA01; s_SOJA01 sp026014845	75.64	2.12	1,319,010	933	1,286
MAG11	d_Archaea; p_Thermoproteota; c_Bathyarchaeia; o_Bathyarchaeales; f_UBA233; g_SOJA01; s_SOJA01 sp030589055	97.34	2.18	2,261,422	1,342	937
MAG12	d_Bacteria; p_Acidobacteriota; c_Aminicenantia; o_Aminicenantales; f_Aminicenantaceae; g_JAUWYH01; s_JAUWYH01 sp03061597	87.66	1.01	2,373,269	1,909	1,648
MAG13	d_Bacteria; p_Acidobacteriota; c_Aminicenantia; o_Aminicenantales; f_Aminicenantaceae; g_SOIV01; s_SOIV01 sp004377005	84.62	0.6	2,046,379	1,560	1,417
MAG14	d_Bacteria; p_Actinomycetota; c_Humimicrobiia; o_Humimicrobiales; f_Humimicrobiaceae; g_Hydromicrobium; s_Hydromicrobium sp004376325	74.90	0.24	993,537	773	841
MAG15	d_Bacteria; p_Actinomycetota; c_Humimicrobiia; o_JAHJRV01; f_JAHJRV01; g_JAHJRV01; s_JAHJRV01 sp030612085	85.74	1.5	1,085,259	871	572
MAG16	d_Bacteria; p_Aerophobota; c_Aerophobia; o_Aerophobales; f_AE-B3A; g_AE-B3B; s_AE-B3B sp005223085	71.99	0.12	1,071,685	1,063	728
MAG17	d_Bacteria; p_Aerophobota; c_Aerophobia; o_Aerophobales; f_AE-B3A; g_SOJT01; s_SOJT01 sp004376485	92.02	4.65	1,768,997	1,685	1,098
MAG18	d_Bacteria; p_Aerophobota; c_Aerophobia; o_Aerophobales; f_AE-B3A; g_SOJT01; s_SOJT01 sp030602105	88.11	2.53	1593934	1,493	954
MAG19	d_Bacteria; p_Atribacterota; c_JS1; o_SB-45; f_34-128; g_34-128	75.00	1.94	1,183,022	897	1,200
MAG20	d_Bacteria; p_Atribacterota; c_JS1; o_SB-45; f_34-128; g_34-128	71.99	1.7	1,666,186	1,216	1,621
MAG21	d_Bacteria; p_Atribacterota; c_JS1; o_SB-45; f_34-128; g_34-128	91.05	1.23	2,164,072	1,555	1,801
MAG22	d_Bacteria; p_Chloroflexota; c_Anaerolineae; o_Anaerolineales; f_E44-bin32; g_E44-bin32	81.75	3.41	3,073,760	2,332	1,960
MAG23	d_Bacteria; p_Chloroflexota; c_Anaerolineae; o_Anaerolineales; f_UBA4823; g_JAUWPO01; s_JAUWPO01 sp030611505	81.67	1.44	2,595,578	2,067	1,906

MAG24	d_Bacteria; p_Chloroflexota; c_Anaerolineae; o_UBA7937; f_UBA7937; g_UBA7937; s_UBA7937 sp030593425	84.00	0.26	2,059,739	1,763	1,123
MAG25	d_Bacteria; p_Chloroflexota; c_Anaerolineae; o_VGOG01; f_VGOG01; g_VGOG01; s_VGOG01 sp030615705	79.89	3.91	2,738,837	2,397	1,702
MAG26	d_Bacteria; p_Chloroflexota; c_Dehalococcoidia; o_Dehalococcoidales; f_DAOWJC01; g_DAOWJC01	87.30	0.93	1,412,408	1,172	1,176
MAG27	d_Bacteria; p_Chloroflexota; c_Dehalococcoidia; o_Dehalococcoidales; f_JAUVZA01; g_JAUVZA01	70.99	1.15	806,935	713	796
MAG28	d_Bacteria; p_Chloroflexota; c_Dehalococcoidia; o_Dehalococcoidales; f_JAUVZE01; g_JAUVZE01	93.11	3.98	2,103,831	1,648	1,547
MAG29	d_Bacteria; p_Chloroflexota; c_Dehalococcoidia; o_Dehalococcoidales; f_RBG-16-60-22; g_E44-bin89; s_E44-bin89 sp004375725	72.55	0.33	1,158,296	1,338	1,234
MAG30	d_Bacteria; p_Chloroflexota; c_Dehalococcoidia; o_Dehalococcoidales; f_RBG-16-60-22; g_JAUVXN01	75.87	0.18	1,339,204	1,048	1,078
MAG31	d_Bacteria; p_Chloroflexota; c_Dehalococcoidia; o_Dehalococcoidales; f_UBA2162	89.92	1.45	1,703,253	1,646	1,370
MAG32	d_Bacteria; p_Chloroflexota; c_Dehalococcoidia; o_E44-bin15; f_E44-bin15	84.85	5.68	1,661,784	1,841	1,365
MAG33	d_Bacteria; p_Chloroflexota; c_Dehalococcoidia; o_E44-bin15; f_E44-bin15; g_E29-bin15; s_E29-bin15 sp004377275	86.41	2.41	1,463,208	1,321	1,171
MAG34	d_Bacteria; p_Chloroflexota; c_Dehalococcoidia; o_E44-bin15; f_E44-bin15; g_E44-bin26; s_E44-bin26 sp030619615	75.98	1.52	936,717	1,012	912
MAG35	d_Bacteria; p_Chloroflexota; c_Dehalococcoidia; o_GIF9; f_AB-539-J10; g_20-14-all-45-10	79.26	2.12	862,419	783	758
MAG36	d_Bacteria; p_Chloroflexota; c_Dehalococcoidia; o_GIF9; f_AB-539-J10; g_B68-G16; s_B68-G16 sp030611015	75.91	1.84	912,201	785	849
MAG37	d_Bacteria; p_Chloroflexota; c_Dehalococcoidia; o_GIF9; f_AB-539-J10; g_JAUWFX01	87.56	2.06	1,445,593	1,377	1,308
MAG38	d_Bacteria; p_Chloroflexota; c_Dehalococcoidia; o_SM23-28-2; f_SM23-28-2; g_SM23-28-2	90.22	3.06	1,992,346	1867	1,622
MAG39	d_Bacteria; p_Chloroflexota; c_Dehalococcoidia; o_SZUA-161; f_SZUA-161; g_SOKG01; s_SOKG01 sp004376205	89.72	2.56	2,000,421	2,119	1,570
MAG40	d_Bacteria; p_Desulfobacterota; c_DTXE01; o_DTXE01; f_DTXE01; g_DTXE01	92.74	4.84	3,002,988	2,337	1,681
MAG41	d_Bacteria; p_Patescibacteria; c_Paceibacteria; o_Paceibacterales	90.02	0.53	416,493	330	439
MAG42	d_Bacteria; p_Patescibacteria; c_Paceibacteria; o_Paceibacterales; f_JAGGXC01	92.63	0.4	794,288	423	529
MAG43	d_Bacteria; p_Patescibacteria; c_Paceibacteria; o_RBG-13-40-8-A	86.82	0.26	453,389	283	403
MAG44	d_Bacteria; p_Planctomycetota; c_Phycisphaerae; o_Sedimentisphaerales; f_SG8-4; g_JAUVYQ01	90.23	1.6	4,146,163	2,264	2,334
MAG45	d_Bacteria; p_Pseudomonadota; c_Alphaproteobacteria; o_Caulobacterales; f_Caulobacteraceae; g_Caulobacter; s_Caulobacter vibrioides_E	75.45	2.56	3,919,226	2,904	3,901
MAG46	d_Bacteria; p_Pseudomonadota; c_Gammaproteobacteria; o_Xanthomonadales; f_Xanthomonadaceae; g_Lysobacter_F; s_Lysobacter_F thermophilum	80.61	0	2,320,604	1,529	2,032
MAG47	d_Bacteria; p_WOR-3; c_WOR-3; o_SM23-42; f_SM23-42; g_SOIY01; s_SOIY01 sp004376785	78.30	1.1	1,614,067	1,038	1,148
MAG48	d_Bacteria; p_Zixibacteria; c_MSB-5A5; o_UBA10806; f_UBA10806	85.06	1.84	2,720,075	1,873	1,735
